# miR-302a-5p/367-3p-HMGA2 axis regulates malignant processes during endometrial cancer development

**DOI:** 10.1186/s13046-018-0686-6

**Published:** 2018-02-01

**Authors:** Jian Ma, Da Li, Fan-Fei Kong, Di Yang, Hui Yang, Xiao-Xin Ma

**Affiliations:** Department of Obstetrics and Gynecology, Key Laboratory of Maternal-Fetal Medicine of Liaoning Province, Key Laboratory of Obstetrics and Gynecology of Higher Education of Liaoning Province, Shengjing Hospital of China Medical University, Shenyang, 110004 China

**Keywords:** miRNAs, HMGA2, Regulation, EMT, Endometrial cancer

## Abstract

**Background:**

Metastasis is one of the main reasons for treatment failure in endometrial cancer. Notably, high mobility group AT-hook 2 (HMGA2) has been recognized as a driving factor of tumour metastasis. microRNAs (miRNAs) are powerful posttranscriptional regulators of HMGA2**.**

**Methods:**

The binding sites of miR-302a-5p and miR-367-3p on HMGA2 mRNA were identified using bioinformatics prediction software and were validated via luciferase assay. The expression levels of miR-302a-5p and miR-367-3p were detected using quantitative real-time PCR and in situ hybridization. Western blotting and immunohistochemistry were used to detect the levels of HMGA2 and epithelial-mesenchymal transition pathway-related proteins. Co-immunoprecipitation was used to detect protein interactions. The roles of miR-302a-5p and miR-367-3p in the regulation of HMGA2 during the progression of endometrial cancer were investigated using both in vitro and in vivo assays.

**Results:**

In the present study, high HMGA2 expression was correlated with poor clinical outcomes in endometrial cancer. The binding sites of miRNAs on HMGA2 mRNA were identified using bioinformatics prediction software and were validated via luciferase assay. In the endometrial cancer cell lines Ishikawa and HEC-1A, the overexpression of miR-302a-5p/367-3p significantly inhibited the expression of HMGA2 mRNA. In endometrial cancer tissues, we showed that miR-302a-5p and miR-367-3p were significantly downregulated and thus inversely correlated with HMGA2. The miR-302a-5p and miR-367-3p expression levels were closely correlated with FIGO stage and lymph node metastasis. High expression of miR-302a-5p/367-3p was correlated with high survival rates in endometrial cancer. In addition, miR-302a-5p/367-3p suppressed the malignant behaviour of endometrial carcinoma cells via the inhibition of HMGA2 expression.

**Conclusion:**

Our findings indicate that miR-302a-5p/367-3p-mediated expression of HMGA2 regulates the malignant behaviour of endometrial carcinoma cells, which suggests that the miR-302a-5p/367-3p-HMGA2 axis may be a predictive biomarker of endometrial cancer metastasis and patient survival and a potential therapeutic target in metastatic endometrial cancer.

**Electronic supplementary material:**

The online version of this article (10.1186/s13046-018-0686-6) contains supplementary material, which is available to authorized users.

## Background

With increasing incidence over the years, endometrial cancer is the most common malignancy of the female reproductive tract in developed countries [1]. Treatment of endometrial cancer includes surgery supplemented with radiotherapy, chemotherapy, and therapy with hormones or targeted therapy such as rapamycin [2]. For patients with advanced metastatic or recurrent endometrial cancer, treatment failure is still high due to the loss of opportunity for surgery. With the elucidation of molecular mechanisms in endometrial cancer, an increasing number of molecular targeted drugs are being tested for clinical application, and some have shown beneficial effects [3, 4]. Therefore, the potential mechanisms in the development of endometrial cancer, especially those involved in tumour metastasis, have become the basis for the development of molecular targeted therapy for endometrial cancer, which is essential for the treatment of this type of cancer.

High mobility group AT-hook 2 (HMGA2) is a chromatin-binding protein that organizes protein complexes on enhancers of various genes to regulate gene expression and cell differentiation [5]. HMGA2 has emerged as a candidate tumour biomarker [6] because it is overexpressed in many cancer patients and is undetectable in adults under normal physiological conditions [7]. In addition, the expression of the HMGA2 is associated with tumour stage and clinical outcomes in ovarian carcinoma [8]. As an oncogene, HMGA2 regulates cell proliferation, the cell cycle and apoptosis, and it is involved in the regulation of invasion and metastasis, which are key processes in tumour progression, such as ovarian tumours [9], breast adenocarcinoma [10] and lung cancers [11]. Until now, it has been reported that HMGA2 is highly expressed in endometrial cancer tissues and that the overexpression of HMGA2 promotes tumour growth and metastasis [12]. However, the mechanisms by which HMGA2 regulates the biological characteristics of endometrial cancer as well as the factors involved remain undefined.

MicroRNAs (miRNAs) are molecules that can regulate a set of target genes and lead to translational repression or mRNA degradation. Recent studies have demonstrated extensive deregulation of miRNAs in endometrial carcinoma, which regulate cell growth and metastasis and affect tumour initiation and development [13]. miRNAs regulating the activity of HMGA2 are considered major molecular mediators in tumour metastasis [14].

The epithelial-mesenchymal transition (EMT) is a central process in the invasion and metastasis of human tumours [15, 16]. HMGA2 is a driving factor of this process [17–19]. Identification of the mechanisms triggering EMT may serve as an efficient strategy for the treatment of malignant and metastatic tumours.

In this study, we identified two novel aberrantly-expressed miRNAs, miR-302a-5p and miR-367-3p, that modulate the malignant behaviour of endometrial carcinoma cells through the suppression of HMGA2 expression.

## Methods

### Bioinformatics prediction

To predict miRNAs with potential binding sites on HMGA2 mRNA, we employed 2 types of bioinformatics software, namely, TargetScan 6.0 (http://www.targetscan.org/) and miRanda (http://www.microrna.org/microrna/home.do). To analyse the clinicopathologic parameters and overall survival curves of the endometrial carcinoma patients with high or low HMGA2 mRNA expression and the relationship between HMGA2 protein and RUNX1 protein levels, data from The Cancer Genome Atlas (TCGA) dataset were retrieved and analysed.

### Patients and samples

Forty endometrial cancer tissues, 37 normal fresh endometrial tissues and 99 cases of paraffin-embedded tissue sections (80 endometrial cancers and 19 normal endometrial tissues) were obtained from the Department of Obstetrics and Gynaecology, Shengjing Hospital of China Medical University from patients undergoing surgical resection from 2011 to 2017, excluding patients with liver and kidney disease and patients without preoperative radiotherapy and chemotherapy. Postoperative histopathology of paraffin sections was used to confirm the diagnosis of endometrial cancer according to the International Union of Obstetrics and Gynaecology (FIGO 2009) staging standards. Samples from normal intimal lining of the uterus at proliferative and secretory phases were used as controls. All protocols were approved by the agency, and consent was received from the patients.

### Cell culture

The endometrial cancer cell lines Ishikawa and HEC-1A were maintained in RPMI 1640 medium (Gibco, Carlsbad, CA, USA) and McCoy’s 5A medium (Gibco) respectively. The human embryonic kidney cell line HEK293T was maintained in high-glucose DMEM (Gibco). The cells were cultured in the presence of 10% foetal bovine serum (FBS) (Gibco), 50 IU/mL penicillin, and 50 mg/mL streptomycin (Invitrogen, Carlsbad, CA, USA). All cells were cultured at 37 °C in a humidified incubator in the presence of 5% CO_2_. All cells were obtained from the Institute of Biochemistry and Cell Biology, Chinese Academy of Sciences (Shanghai, China).

### Transfection of cells

The overexpression plasmid (gV144-HMGA2), knockdown plasmid (sh-HMGA2) and their respective negative control (NC) counterparts were purchased from GeneChem (Shanghai, China). Agomir, antagomir and scrambled negative control RNAs were purchased from GenePharma (Shanghai, China). Lipofectamine 3000 (Invitrogen) was used to transfect cells with miRNAs and HMGA2 for subsequent experiments according to the manufacturer’s instructions. The sequences of the shRNA clone and the agomir and antagomir are listed in Additional file 1: Table S1.

### RNA extraction and quantitative RT-PCR

RNA was extracted from tissue or cells with TRIzol (Takara, Dalian, China). After measurement of the RNA concentration, cDNAs were generated from reverse transcription of RNA. cDNAs corresponding to the mRNAs of interest were reverse transcribed using PrimeScript RT-polymerase (Takara). cDNAs corresponding to the miRNAs of interest were synthesized using Mir-X miRNA First-Strand Synthesis kit (Clontech, Dalian, China), and SYBR Green Premix (Takara) was used to perform quantitative real-time PCR (qRT-PCR) according to the manufacturer’s instructions. The specific PCR primers were designed by Sangon Biotech Co. Ltd. (Shanghai, China). With GAPDH and U6 as internal controls, fold-changes were calculated using the 2^-∆∆Ct^ method. The primer sequences are listed in Additional file 2: Table S2.

### Protein extraction, western blotting and co-immunoprecipitation assay

Total protein was extracted with the Protein Extraction kit (Beyotime Biotechnology, Shanghai, China). Proteins were separated using 10% sodium dodecyl sulphate (SDS)-polyacrylamide gel electrophoresis (PAGE) and then transferred to polyvinylidene difluoride (PVDF) membranes (Millipore, MA, USA). Then, the membranes were incubated with primary antibodies overnight at 4 °C (the primary antibodies are listed in Additional file 3: Table S3) and subsequently incubated with secondary antibodies for 1.5 h at room temperature. After being washed, the membranes were visualized using Quantity One imaging software (Bio-Rad, CA, USA). For co-immunoprecipitation experiments, ice-cold RIPA buffer was added to the cells. After centrifugation, the supernatant fractions were collected and treated with an anti-HMGA2 antibody for 3 h at 4 °C. Protein A/G PLUS-Agarose (Santa Cruz Biotechnology, Santa Cruz, CA, USA) was then added, followed by overnight incubation at 4 °C on a rocker platform. Then, the samples were processed following the protocols for western blotting. The intensities of the protein bands were quantified using ImageJ software v.1.48 [http://imagej.nih.gov/ij/download.html].

### Enzyme-linked immunosorbent assay

The levels of matrix metalloproteinase-2 (MMP-2) and matrix metalloproteinase-9 (MMP-9) in the conditioned medium were quantified using Quantikine ELISA kits (R&D systems, Minneapolis, USA) per the manufacturer’s guidelines.

### Luciferase assay

HEK293T cells were plated in 96-well plates. The cells were co-transfected with miRNAs or scrambled NC (RiboBio, Guangzhou, China) and 100 ng of wild-type or mutant dual-luciferase reporter vector containing an HMGA2 gene fragment (pmiR-RB-Report-HMGA2) (RiboBio) using Lipofectamine 3000. After 48 h of cell transfection, the luciferase activity was measured using the Dual-Luciferase Reporter Assay System (Promega, Madison, WI, USA). The experiments were performed in triplicate.

### Cell proliferation assay

Cells were seeded in 96-well plates, and 10 μL CCK-8 (Cell Counting Kit-8; Dojindo, Japan) reagent was added per well. After incubation for 3 h at 37 °C in 5% CO_2_, the OD at 450 nm of each well was measured with a microplate reader at 24 h, 48 h and 72 h. We detected cell proliferation with an EdU Cell Proliferation Assay kit (RiboBio). Cells were incubated with 50 μM EdU for 2 h, and then to stain the DNA, 100 μL of 1 μg/mL Hoechst 3342 reaction mixture was added to each well for 30 min. The cells were imaged using a fluorescence microscope (Nikon, Japan). Original magnification: × 20. The experiments were performed in triplicate.

### Wound healing assay

Cells were grown to approximately 90% confluency in 6-well plates, and a straight wound was artificially created on the cell monolayer with a 100-μL pipette tip. The cells were washed with serum-free medium. The ability of cells to migrate to the site of the wound was observed under the microscope at daily intervals, and photographs were captured at 0 h and 48 h. The experiments were performed in triplicate**.** Original magnification: × 10.

### Cell invasion assay

The transwell method was used to detect cell invasion. Transwell filter inserts (8-μM pore size; Corning, NY, USA) were pre-coated with Matrigel for 30 min at 37 °C and washed with serum-free medium. Cells were suspended in 200 μL serum-free medium and placed in the upper chambers, and the lower chambers were filled with 700 μL of culture medium containing 10% FBS as the chemo attractant. After several hours, the non-invading cells on the upper surface of the membranes were wiped off with cotton swabs, and 4% paraformaldehyde was used to fix the invading cells for 50 min, which was followed by staining with 0.1% crystal violet and washing. The invaded cells were quantified via imaging under an inverted fluorescence microscope equipped with an image acquisition system (Nikon, Japan). The experiments were performed in triplicate. Original magnification: × 20.

### Apoptosis and cell cycle analyses

Cellular apoptosis was detected via flow cytometry. Cells were transfected for 48 h, and 10^6^ cells were harvested per group. The cells were washed and stained using an apoptosis detection kit for double staining with Annexin V-APC/7-AAD (KeyGen Biotech, Nanjing, China). After incubation for 15 min, the cells were analysed using flow cytometry (BD FACSCalibur, NJ, USA). Cell cycle distribution was analysed via flow cytometry. After transfection, the cells were harvested, washed and fixed with 70% ethanol at 4 °C overnight. The cells were mixed with 100 μL of RNase, and cellular DNA was stained using PI. The cells were analysed via flow cytometry (BD.

FACSCalibur), and the results were compared with those of cell proliferation assays. The experiment was performed in triplicate.

### Tumour xenografts in nude mice

Tumourigenesis experiments in nude mice were performed in strict accordance with the protocol approved by the Scientific Research and New Technology Ethical Committee of the Shengjing Hospital of China Medical University. Four- to six-week-old (BALB/c) athymic nude mice were purchased from HFK Bioscience Co., Ltd. (Beijing, China). Cells were harvested, suspended at 10^6^ cells/100 μL of PBS and subcutaneously injected into the left axilla of the mice. For treatment, the respective agents were injected into tumours at multiple sites every 4 days. Tumour volume was measured every 4 days, and the mice were sacrificed after 28 days. The excised tumours were fixed at 4 °C in paraformaldehyde and embedded in paraffin. Tumour sections were used for immunohistochemistry analyses. Each group contained three mice.

### Immunohistochemistry and in situ hybridization

Subcutaneous tumours were excised, fixed in 4% paraformaldehyde, dehydrated, embedded in paraffin and sectioned for immunohistochemical analyses. For in situ hybridization (ISH), the digoxigenin-labeled oligonucleotide miR-302a-5p, miR-367-3p detection probe (Boster Biological Technology Co., Ltd., Nanjing, China) was used, and the miR-302a-5p probe sequence was as follows: (5′-3′) AGCAAGTACATCCACGTTTAAGT, miR-367-3p (5′-3′) TCACCATTGCTAAGTGCAATT. The experiments were performed according to the in situ hybridization test kit instructions. The immunostained sections were observed under an optical microscope and scored according to the percentage of positive cells, as follows: less than 10% positive cells, 1; 10% to 50% positive cells, 2; and 51 to 75% positive cells, 3. The staining intensity was scored as weak, 1; medium, 2; and strong, 3. The final score was calculated by multiplying the score for staining intensity with the score corresponding to the percentage of positive cells. Thus, a total score of 1 to 9 was calculated, as follows: ≤ 2 points as negative, 3 ~ 4 as weak positive (+), 5 ~ 8 as moderate positive (+ +), and 9 as strong positive (+ + +). - /+ indicated low expression, ++ /+++ indicated high expression. Original magnification: × 400.

### Statistical analysis

The data were presented as the mean ± SEM of 3 independent experiments. All statistical analyses were performed with GraphPad Prism 6.0 (La Jolla, CA) and SPSS 17.0 software (Abbott Laboratories, Chicago, IL). Student’s *t* test and the Chi-square test were used for comparisons between two independent groups, and one-way ANOVA was used to compare differences among more than two groups. Pearson’s and Spearman’s correlation coefficient analyses were used for the determination of correlation. Survival curves were plotted according to the Kaplan–Meier method and were compared using the log-rank test. Disease-free survival was defined as the time to progression or last follow-up or death from the date of diagnosis. *P* < 0.05 was defined as statistically significant; *P* > 0.05 indicated non-significance (NS).

## Results

### High HMGA2 expression correlates with poor clinical outcomes in endometrial cancer

**Fig. 1:**
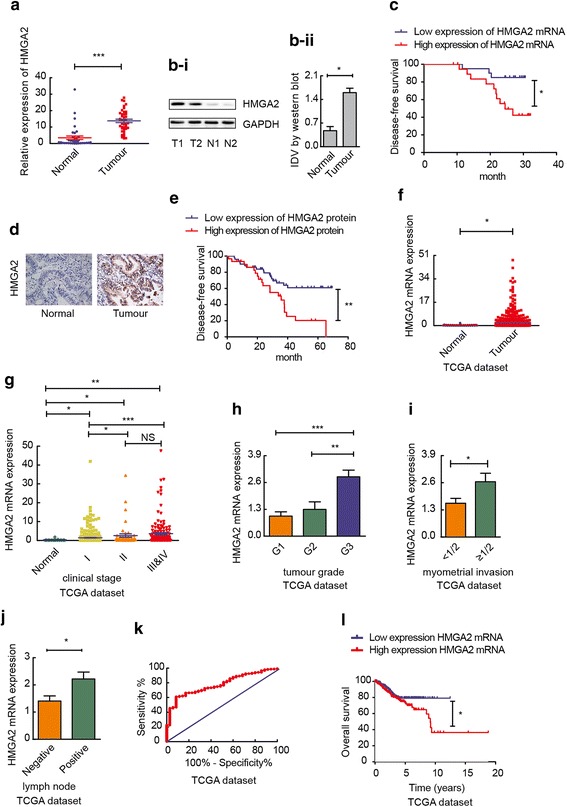
High expression of HMGA2 correlates with worse clinical outcomes in endometrial cancer patients. **a** and **b** HMGA2 was up-regulated in endometrial carcinoma tissues (*n* = 40) compared with normal tissues (*n* = 37). Data are presented as the mean ± SEM. **c** Disease-free survival curves for HMGA2 mRNA in 40 endometrial carcinoma cases. **d** The expression of HMGA2 was detected via immunohistochemistry in endometrial cancer (*n* = 80) and normal endometrial tissue (*n* = 19). **e** Disease-free survival curves for HMGA2 protein in 80 endometrial carcinoma cases. **f** Compared with normal endometrial tissue (*n* = 35), (the controls were collected from paracancerous tissues in patients with endometrial cancer). HMGA2 was highly expressed in 552 endometrial carcinoma samples (TCGA cohort). **g** HMGA2 expression levels in patients with different clinical stages of endometrial cancer (TCGA cohort): normal (*n* = 35), I (*n* = 339), II (*n* = 48), III & IV (*n* = 153). **h** HMGA2 expression levels in patients with endometrial cancers of different grades (TCGA cohort): G1 (*n* = 109), G2 (*n* = 120), G3 (*n* = 314). **i** Myometrial invasion (TCGA cohort): < 1/2 group (*n* = 259), ≥ 1/2 group (*n* = 211). **j** Lymph node status (TCGA cohort): negative group (*n* = 190), positive group (*n* = 322). **k** ROC of HMGA2 (TCGA cohort). **l** High expression of HMGA2 predicted a shorter overall survival in endometrial cancer. The data were retrieved and analysed from the TCGA dataset (*n* = 542). *P < 0.05, **P < 0.01, ***P < 0.0001

The results of qRT-PCR and western blotting showed that the expression level of HMGA2 was higher in endometrial carcinoma tissues than that in normal endometrial tissues (Fig. 1a and b). The immunohistochemistry results showed that the positive rate of HMGA2 protein expression in endometrial cancer was 80.0%, which was much higher than that of normal endometrial tissue (10.5%) (Fig. 1d, Additional file 4: Table S4-i). In addition, we analysed the association of the levels of HMGA2 protein (Additional file 4: Table S4-ii) and mRNA (Additional file 5: Table S5) with the clinicopathologic parameters and the disease-free survival of endometrial cancer patients. The results showed that HMGA2 expression was significantly associated with the clinicopathological features. HMGA2 was increased in the progression from stage I to stages III & IV. In addition, we found that HMGA2 expression was significantly associated with tumour grade and myometrial invasion in patients with endometrial cancer and that HMGA2 expression levels were significantly up-regulated in the tissues of endometrial cancer patients with lymph node metastasis compared with those of patients without lymph node metastasis. The disease-free survival curves for the endometrial carcinoma patients with high or low HMGA2 mRNA (Fig. 1c) and protein expression (Fig. 1e) indicated that high expression of HMGA2 correlates with poor clinical outcomes in endometrial cancer. Based on the TCGA dataset, HMGA2 showed a dramatic overexpression in endometrial cancer tissue compared with that in normal endometrial tissues (Fig. 1f). Moreover, based on the TCGA cohort, we analysed the association between the levels of HMGA2 and the clinicopathologic parameters of endometrial cancer patients. We found that HMGA2 expression was significantly associated with clinical stage, differentiation, infiltration depth and lymphatic metastasis (Fig. 1g-j). Then, we examined the sensitivity and specificity of HMGA2. A receiver operating characteristic (ROC) curve analysis was performed, and the correlation area under the curve (AUC) was used to confirm the diagnostic efficacy of HMGA2. As shown in Fig. 1k, the AUC of HMGA2 reached 0.7761, and the cut-off value was 0.4121, (95% CI: 0.7140 - 0.8382). These results suggest that HMGA2 can discriminate between endometrial carcinoma and normal endometrial tissue. To analyse the overall survival curves for the endometrial carcinoma patients in reference to HMGA2 mRNA expression, we retrieved and analysed the data from the TCGA dataset. The results showed that high expression of HMGA2 correlates with a lower survival rate in endometrial cancer (Fig. 1l).

### Knockdown of HMGA2 inhibits the malignant behaviours of Ishikawa and HEC-1A cell lines

**Fig. 2:**
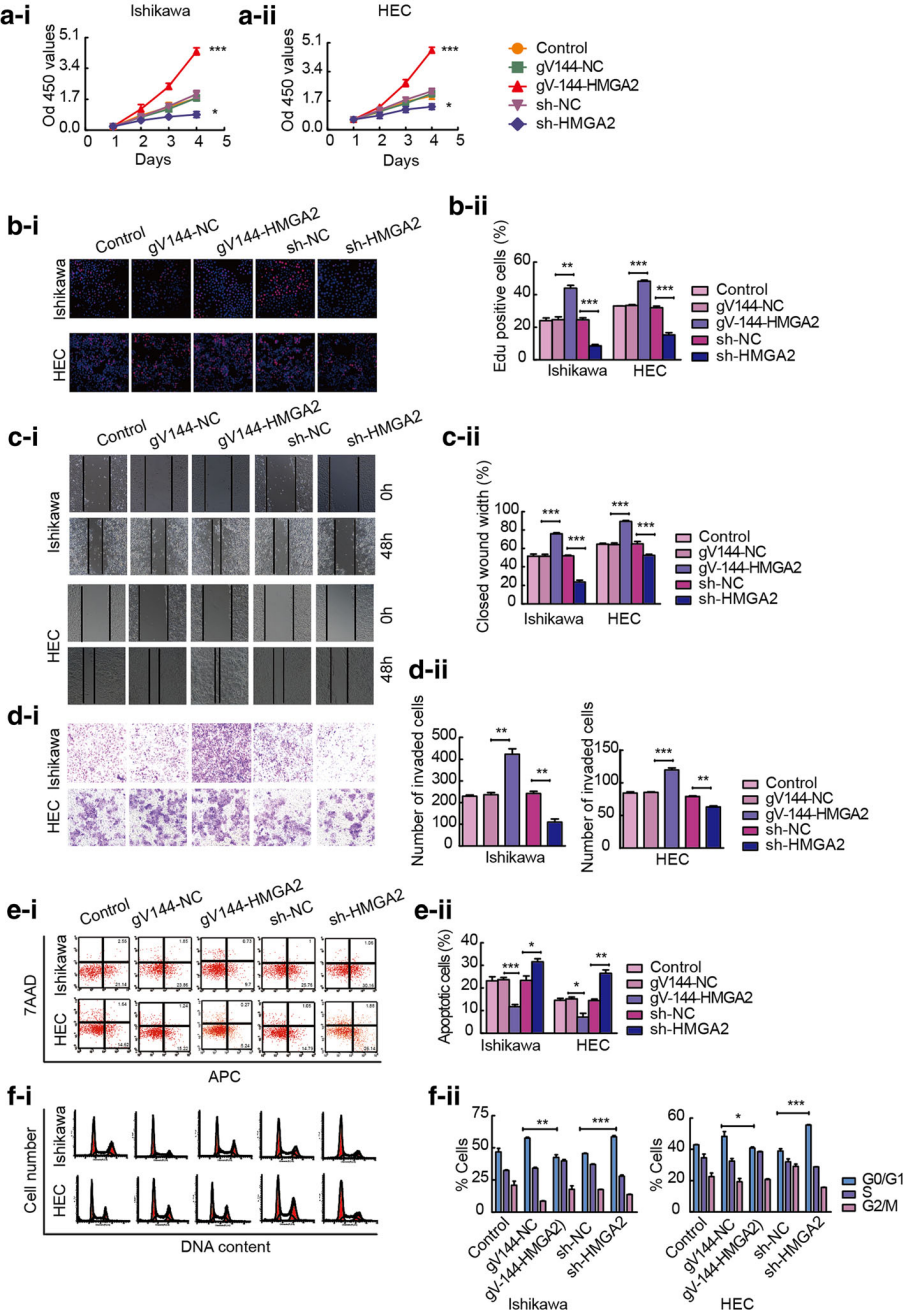
HMGA2 plays an oncogenic role in Ishikawa and HEC-1A cell lines. **a** and **b** CCK-8 and EdU assays were used to detect the effect of HMGA2 expression on the proliferation of Ishikawa and HEC-1A cells. **c** Effects of different levels of HMGA2 on the migration of Ishikawa and HEC-1A cells were evaluated using a wound healing assay. **d** Transwell assays were used to detect cellular invasion. Representative images and accompanying statistical plots are presented. **e** The effects of different levels of HMGA2 on apoptosis were verified via flow cytometry. **f** Effect of HMGA2 on cell cycle distribution in Ishikawa and HEC-1A cell lines. Data are presented as the mean ± SEM (*n* = 3 per group), *P < 0.05, **P < 0.01, *** *P* < 0.0001 vs. the NC group

In order to investigate the carcinogenic effect of HMGA2 in endometrial carcinoma, we determined the baseline expression of HMGA2 in four endometrial cancer cell lines by qRT-PCR (Additional file 6: Figure S1a). HEC-1A cells exhibited the lowest expression, while Ishikawa cells exhibited the highest expression. We therefore transfected the HMGA2 plasmid into these two cell lines. qRT-PCR and western blot analyses were used to determine the transfection efficiency of HMGA2 (Additional file 6: Figure S1b and c). The effect of HMGA2 on the proliferation of the endometrial cancer cell lines Ishikawa and HEC-1 was studied using CCK-8 and EdU assays after overexpressing or knocking down HMGA2. The proliferation of cells in the sh-HMGA2-transfected group was lower than that in the sh-NC-transfected group. Additionally, the proliferation of cells in the HMGA2-overexpression group was significantly higher than that in the control group (Fig. 2a and b). Furthermore, the ability of the cells to migrate and invade was investigated, and this ability was lower in the sh-HMGA2-transfected group than that in the sh-NC group (Fig. 2c and d). Flow cytometry was used to detect apoptosis. The rate of apoptosis in the sh-HMGA2-transfected group was higher than that in the sh-NC group, and it was reduced in response to the overexpression of HMGA2 (Fig. 2e). Cell cycle distribution was analysed using flow cytometry. HMGA2 knockdown induced cell cycle arrest in the G0/G1 phase (Fig. 2f). Thus, knockdown of HMGA2 exerts tumour-suppressive effects on Ishikawa and HEC-1A cells, while overexpression of HMGA2 promotes the malignant behaviours of these cells, indicating the role of HMGA2 as an oncogene in endometrial cancer.

### The expression of miR-302a-5p/367-3p is low in endometrial carcinoma tissues, and HMGA2 is a target of miR-302a-5p and miR-367-3p

**Fig. 3:**
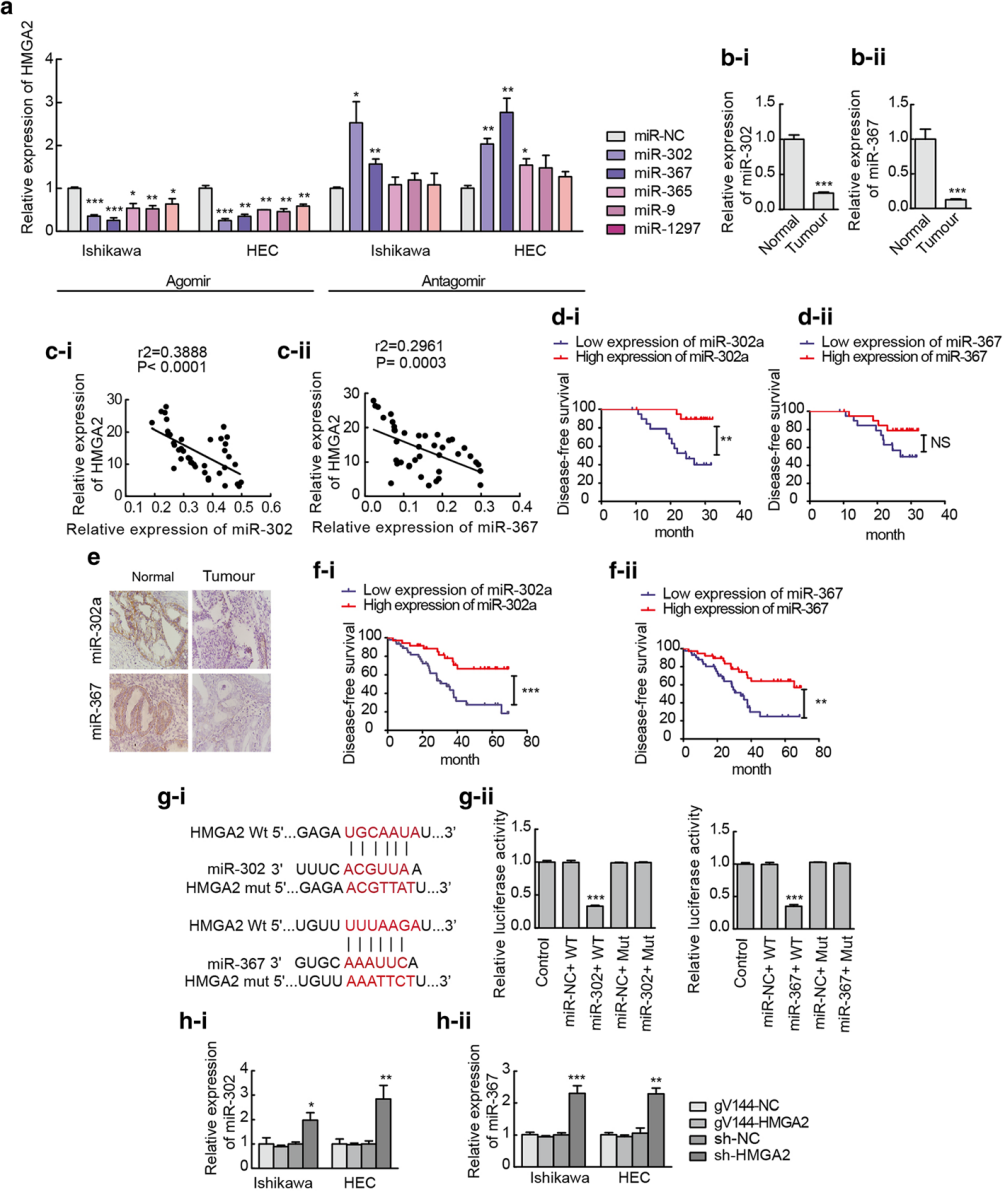
HMGA2 is a target of miR-302a-5p and miR-367-3p. **a** Changes in miRNA expression and the expression of HMGA2 mRNA were detected via qRT-PCR in Ishikawa and HEC-1A cell lines. Data are presented as the mean ± SEM (n = 3 per group). **b** miR-302a-5p/367-3p was downregulated in endometrial carcinoma tissues (n = 40) compared with normal tissues (n = 37). Data are presented as the mean ± SEM. **c** A significant correlation between the levels of miR-302a-5p/367-3p and HMGA2 in endometrial carcinoma tissues (n = 40) was demonstrated using Pearson’s correlation coefficient analysis. miR-302a-5p and HMGA2 (r^2^ = 0.3888, *P* < 0.0001); miR-367-3p and HMGA2 (r^2^ = 0.2961, *P* = 0.0003). **d** Disease-free survival curves for miR-302a-5p/367-3p in 40 endometrial carcinoma tissues (n = 40). NS, *P* > 0.05. **e** and **f** Expression of miR-302a-5p/367-3p in 80 endometrial carcinoma tissues and disease-free survival curves. **g** Predicted miR-302a-5p/367-3p binding sites in the 3’-UTR of wild-type (HMGA2-3’-UTR-WT) and mutant (HMGA2-3’UTR-Mut) HMGA2 sequences. Luciferase reporter assay using HEK293T cells co-transfected with HMGA2-WT (or HMGA2-Mut) and the indicated miRNA. Data are presented as the mean ± SEM (*n* = 3 per group). **h** Expression of miR-302a-5p/367-3p was detected using qRT-PCR after the expression of HMGA2 was altered in Ishikawa and HEC-1A cell lines. Data are presented as the mean ± SEM. (n = 3 per group). *P < 0.05, ** *P* < 0.01, ***P < 0.0001 vs. the NC group

TargetScan and miRanda bioinformatics databases were used to predict miRNAs that can bind to HMGA2. In total, 25 miRNAs were identified, which were screened via co-transfection of HEK293T cells with wild-type dual-luciferase vector-mediated HMGA2 constructs. A significant decrease in luciferase activity was observed in response to transfection with miR-302a-5p, miR-1297, miR-367-3p, miR-365a-3p and miR-9-5p (Additional file 7: Figure S2). Furthermore, when miR-302a-5p, miR-367-3p, miR-365a-3p, miR-9-5p and miR-1297 were overexpressed in Ishikawa and HEC-1A cells, the expression level of HMGA2 was decreased most obviously after miR-302a-5p/367-3p overexpression. Conversely, when cells were transfected with the antagomir-302a-5p/367-3p, HMGA2 expression significantly increased (Fig. 3a), which indicates that HMGA2 expression is likely regulated by miR-302a-5p/miR-367-3p. qRT-PCR was used to determine the transfection efficiency (Additional file 8: Figure S3). In tissues derived from 40 cases of endometrial cancer, the results of qRT-PCR showed that the expression of miR-302a-5p/367-3p in endometrial carcinoma was lower than that in the normal endometrium (Fig. 3b). Moreover, the expression of miR-302a-5p/367-3p was negatively correlated with the expression of HMGA2 (Pearson’s rank correlation method; miR-302a-5p (r^2^ = 0.3888, *P* < 0.0001) and miR-367-3p (r^2^ = 0.2961, *P* = 0.0003) (Fig. 3c). In addition, we analysed the association of the levels of miR-302a-5p/367-3p with the clinicopathologic parameters (Additional file 9: Table S6; Additional file 10: Table S7) and the disease-free survival (Fig. 3d) of endometrial cancer patients. Furthermore, the expression of miR-302a-5p and miR-367-3p in 80 cases of endometrial carcinoma and in 19 normal endometrial tissues was detected by ISH assay. The results showed that the positive rate of miR-302a-5p expression in endometrial cancer was 43.75%, which was much lower than that in normal endometrial tissue (84.2%) (Fig. 3e, Additional file 11: Table S8-i); the positive rate of miR-367-3p expression in endometrial cancer was 47.5%, which was lower than that in normal endometrial tissue (84.2%) (Fig. 3e, Additional file 12: Table S9-i). Additionally, the correlations between the expression of miR-302a-5p/367-3p and HMGA2 protein in endometrial cancer patients were analysed. Spearman’s rank correlation analysis found that the expression of miR-302a-5p/367-3p and the expression of HMGA2 in endometrial cancer were significantly negatively correlated miR-302a-5p: (*r* = − 0.4316, *P* < 0.0001, Additional file 11: Table S8-ii); miR-367-3p: (*r* = − 0.2700, *P* = 0.0154, Additional file 12: Table S9-ii). We also analysed the association of the levels of miR-302a-5p/367-3p with the clinicopathologic parameters (Additional file 11: Table S8-iii; Additional file 12: Table S9-iii) and the disease-free survival (Fig. 3f) of endometrial cancer patients. The results indicated that miR-302a-5p/367-3p expression is associated with muscular invasion and metastasis in patients with endometrial cancer and that high expression of miR-302a-5p/367-3p is correlated with high survival rates in endometrial cancer. The dual-luciferase gene reporter assay demonstrated that miR-302a-5p/367-3p could bind to HMGA2 at the predicted binding sites (Fig. 3g). Interestingly, the expression of miR-302a-5p/367-3p was increased after the HMGA2 gene was knocked down (Fig. 3h), Thus, HMGA2 is regulated by miR-302a-5p/367-3p not only in a unidirectional manner but also in the form of a regulatory feedback loop. Hence, we hypothesize the existence of a regulatory feedback mechanism between miR-302a-5p/miR-367-3p and HMGA2.

### Overexpression of miR-302a-5p/367-3p inhibits cell proliferation, migration, and invasion and promotes apoptosis and cell cycle arrest

**Fig. 4:**
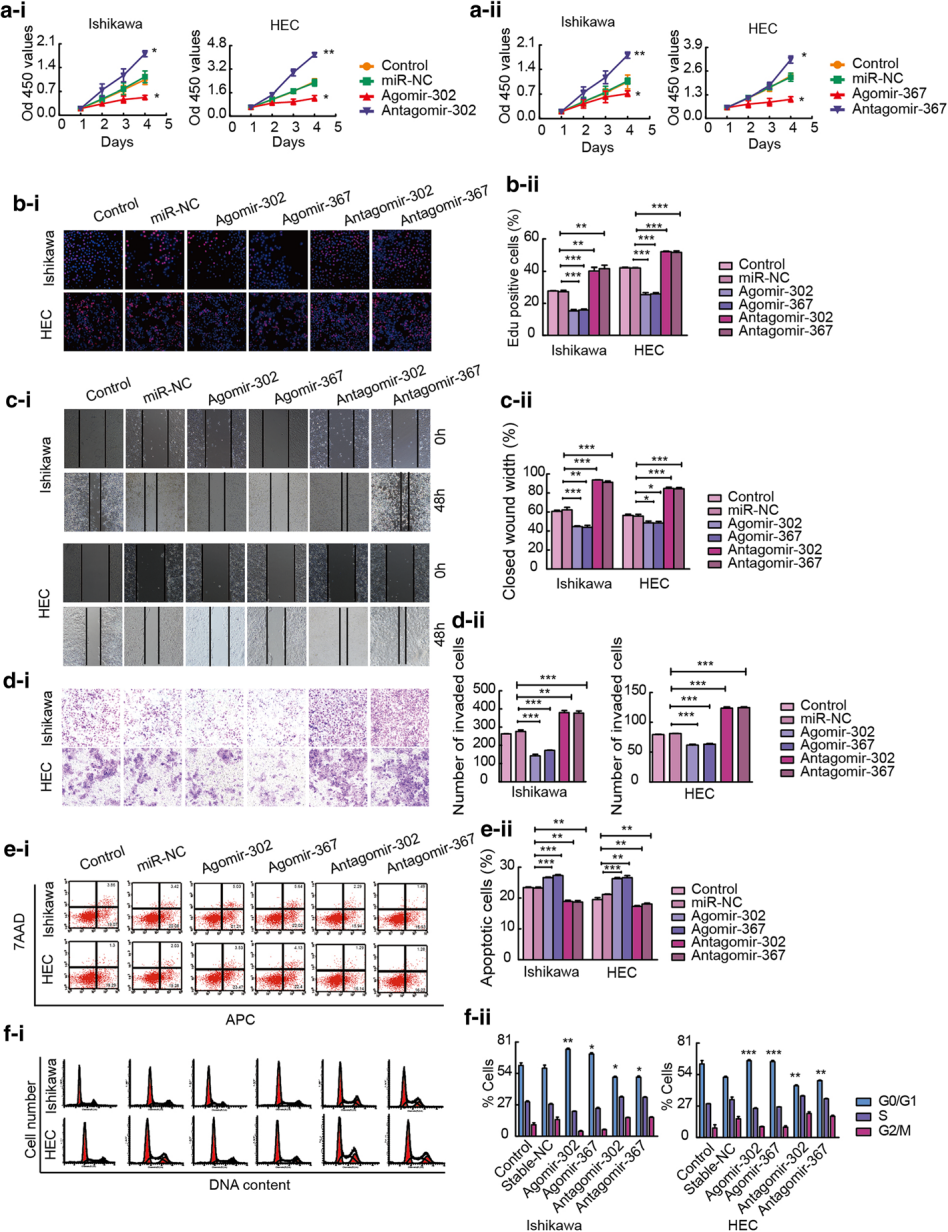
miR-302a-5p/367-3p inhibits malignant biological behaviour of Ishikawa and HEC-1A cell lines. **a** and **b** CCK-8 and EdU assays were used to evaluate effect of miR-302a-5p/367-3p on the proliferation Ishikawa and HEC-1A cell lines. **c** Representative images of the wound healing assay in Ishikawa and HEC-1A cell lines in response to changes in the expression of miR-302a-5p/367-3p. **d** Transwell assays were used to detect the effect of miR-302a-5p/367-3p expression on invasion in Ishikawa and HEC-1A cell lines. Representative images and accompanying statistical plots are presented. **e** Effects of miR-302a-5p/367-3p on apoptosis in Ishikawa and HEC-1A cell lines. **f** Effects of miR-302a-5p/367-3p on cell cycle distribution in Ishikawa and HEC-1A cell lines. Data are presented as the mean ± SEM (*n* = 3 per group). *P < 0.05, ** *P* < 0.01, *** *P* < 0.0001 vs. the miR-NC group

To explore the possible biological significance of miR-302a-5p/367-3p in endometrial cancer, Ishikawa and HEC-1A cells were transfected with agomir-302a-5p/367-3p or antagomir-302a-5p/367-3p, and changes in proliferation, invasion, apoptosis and cell cycle distribution were detected. Cell proliferation was assessed using CCK-8 and EdU assays. Transfection of cells with agomir-302a-5p/367-3p decreased cell proliferation compared with that of transfection with miR-NC, but cell proliferation was promoted after transfection of cells with antagomir-302a-5p/367-3p (Fig. 4a and b). Wound healing and transwell assays were used to evaluate cell migration and invasion, respectively. The migratory and invasion abilities of Ishikawa and HEC-1A cells were reduced in response to the overexpression of miR-302a-5p/367-3p (Fig. 4c and d). Flow cytometry was used to detect the rate of apoptosis in these cells. The overexpression of miR-302a-5p/367-3p promoted apoptosis, whereas the knockdown of miR-302a-5p/367-3p inhibited apoptosis (Fig. 4e). Flow cytometry was used to analyse cell cycle distribution. Cells transfected with agomir-302a-5p/367-3p were arrested at the G0/G1 phase (Fig. 4f).

### HMGA2 is a functional target of miR-302a-5p/367-3p in the regulation of EMT-associated proteins in Ishikawa and HEC-1A cell lines

**Fig. 5:**
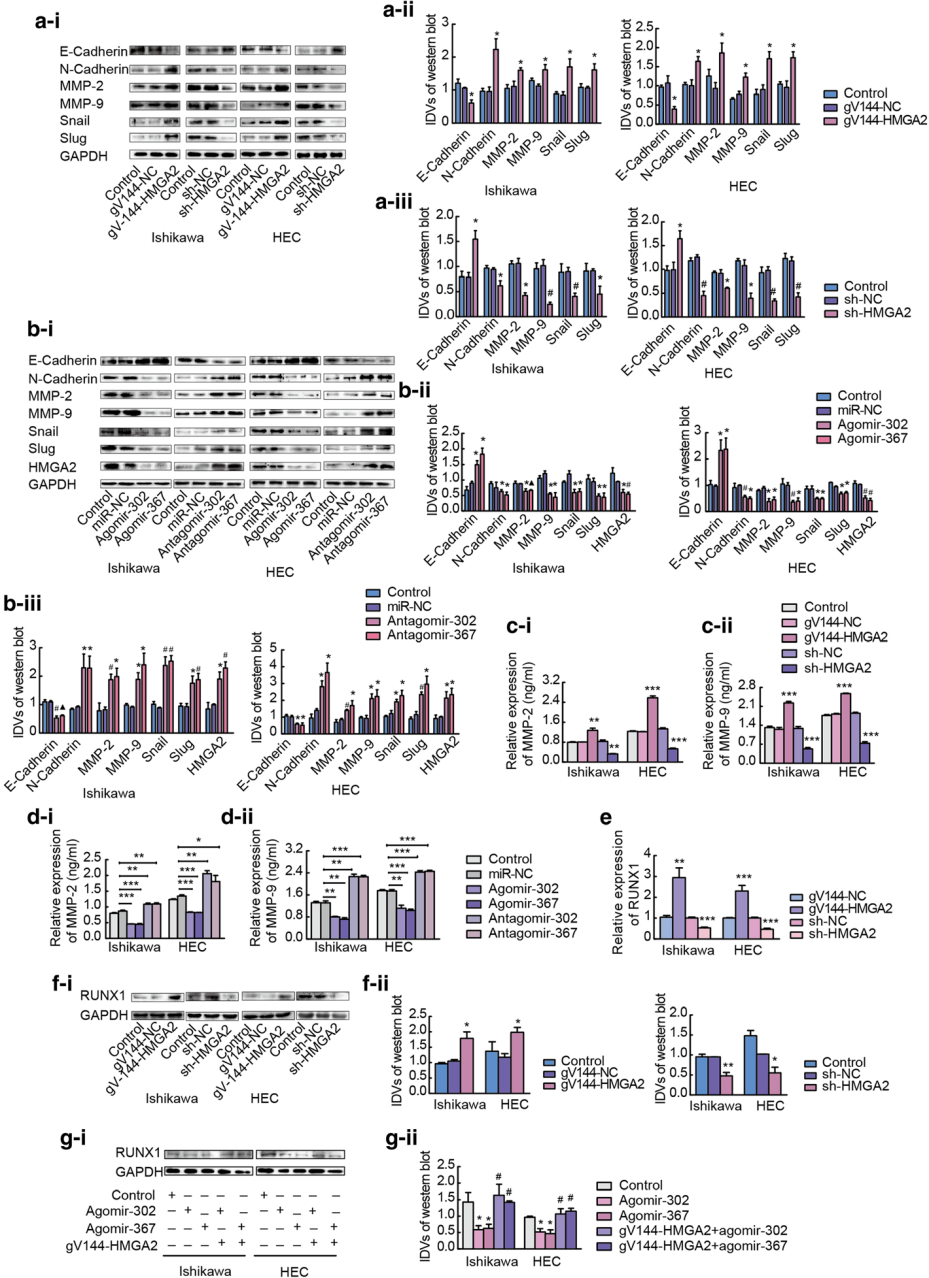
HMGA2 is a functional target of miR-302a-5p/367-3p in regulating EMT. **a** Western blot analysis of the expression of EMT-related proteins including E-cadherin, N-cadherin, MMP-2, MMP-9, Snail and Slug in response to HMGA2 in Ishikawa and HEC-1A cell lines. Relative expression of GAPDH as an endogenous control. Data are presented as the mean ± SEM, (*n* = 3 per group). **b** Western blot analysis of the expression of EMT-related protein including E-cadherin, N-cadherin, MMP-2, MMP-9, Snail and Slug in response to miR-302a-5p/367-3p. Data are presented as the mean ± SEM. (*n* = 3 per group), *P < 0.05, # *P* < 0.01, ▲ *P* < 0.0001. **c** and **d** Concentrations of MMP-2 and MMP-9 proteins in the supernatant of Ishikawa and HEC-1A cell cultures were detected by ELISA *P < 0.05, ** *P* < 0.01, *** *P* < 0.0001, (n = 3 per group). **e** and **f** qRT-PCR and western blotting of HMGA2-regulated RUNX1 expression in Ishikawa and HEC-1A cell lines. Data are presented as the mean ± SEM. *P < 0.05, ** *P* < 0.01, *** *P* < 0.0001 vs. the NC group, (n = 3 per group). **g** Western blot analysis of RUNX1 protein levels in Ishikawa and HEC-1A cell. Data are presented as the mean ± SEM. *P < 0.05, ** *P* < 0.01, *** *P* < 0.0001 vs. the NC group, (*n* = 3 per group)

To further study the mechanism by which miR-302a-5p/367-3p and HMGA2 regulate cell migration and invasion, we examined the levels of EMT-associated proteins in Ishikawa and HEC-1A cells. The effects of HMGA2, miR-302a-5p and miR-367-3p on EMT-related proteins were detected using western blotting. Knockdown of HMGA2 expression or overexpression of miR-302a-5p/367-3p decreased the expression of EMT-related proteins including Snail, Slug, N-cadherin, MMP-2, and MMP-9 and increased the expression of E-cadherin (Fig. 5a and b). In addition, the concentrations of MMP-2 and MMP-9 in the supernatant of Ishikawa and HEC-1A cell cultures were detected by Enzyme-linked immunosorbent assay (ELISA). The results showed that the knockdown of HMGA2 expression or the overexpression of miR-302a-5p/367-3p decreased the concentration of the MMP-2 and MMP-9 proteins in the cell culture medium, which suggests that interference in HMGA2 expression or the high expression of miR-302a-5p/367-3p inhibits Ishikawa and HEC-1A cell secretion of MMP-2 and MMP-9 proteins (Fig. 5c and d). Therefore, overexpression of miR-302a-5p/367-3p or knockdown of HMGA2 reversed EMT in endometrial cancer cells. In other words, HMGA2 is a functional target of miR-302a-5p/367-3p in the regulation of EMT-associated proteins in Ishikawa and HEC-1A cell lines.

### miR-302a-5p/367-3p, HMGA2 and RUNX1 form a regulatory axis in endometrial carcinoma cells

The results of qRT-PCR and the TCGA dataset analysis showed that the expression level of RUNX1 was higher in endometrial carcinoma tissues than that in normal endometrial tissues (Additional file 13: Figure S4a and b). Moreover, the expression of RUNX1 was positively correlated with the expression of HMGA2 in endometrial carcinoma tissue (Additional file 13: Figure S4c and d). To further study the mechanism by which RUNX1 regulate cell migration and invasion, we examined the expression of MMP-2 and MMP-9 in Ishikawa and HEC-1A cells. The results showed that overexpressed of RUNX1 increased the expression of MMP-2 and MMP-9 at the mRNA and protein levels (Additional file 13: Figure S4e and f). In addition, the results of ELISA showed that the overexpressed of RUNX1 increased the concentration of the MMP-2 and MMP-9 proteins in the cell culture medium (Additional file 13: Figure S4 g). Co-immunoprecipitation assays were performed in Ishikawa and HEC-1A cells. HMGA2 was found to be bound to RUNX1 (Additional file 13: Figure S4 h). To verify whether RUNX1 is involved in the regulation of HMGA2, HMGA2 was overexpressed or knocked down in Ishikawa and HEC-1A cells, and qRT-PCR and western blot analyses were performed to detect the mRNA and protein expression levels of RUNX1. Overexpression of HMGA2 increased the expression of RUNX1 at the mRNA and protein levels (Fig. 5e and f). We then investigated whether miR-302a-5p/367-3p modulates RUNX1 expression through regulation of HMGA2. First, we screened the full RUNX1 mRNA sequence from TargetScan bioinformatics databases and identified no miR-302a-5p/367-3p binding sites. Then, Ishikawa and HEC-1A cells were co-transfected with overexpression constructs of miR-302a-5p/367-3p and HMGA2. Notably, rescue experiments demonstrated that miR-302a-5p/367-3p-dependent downregulation of RUNX1 protein was largely negated by overexpression of HMGA2 (Fig. 5g).

### miR-302a-5p/367-3p controls the HMGA2-mediated suppression of the malignant behaviour of endometrial carcinoma cells

**Fig. 6:**
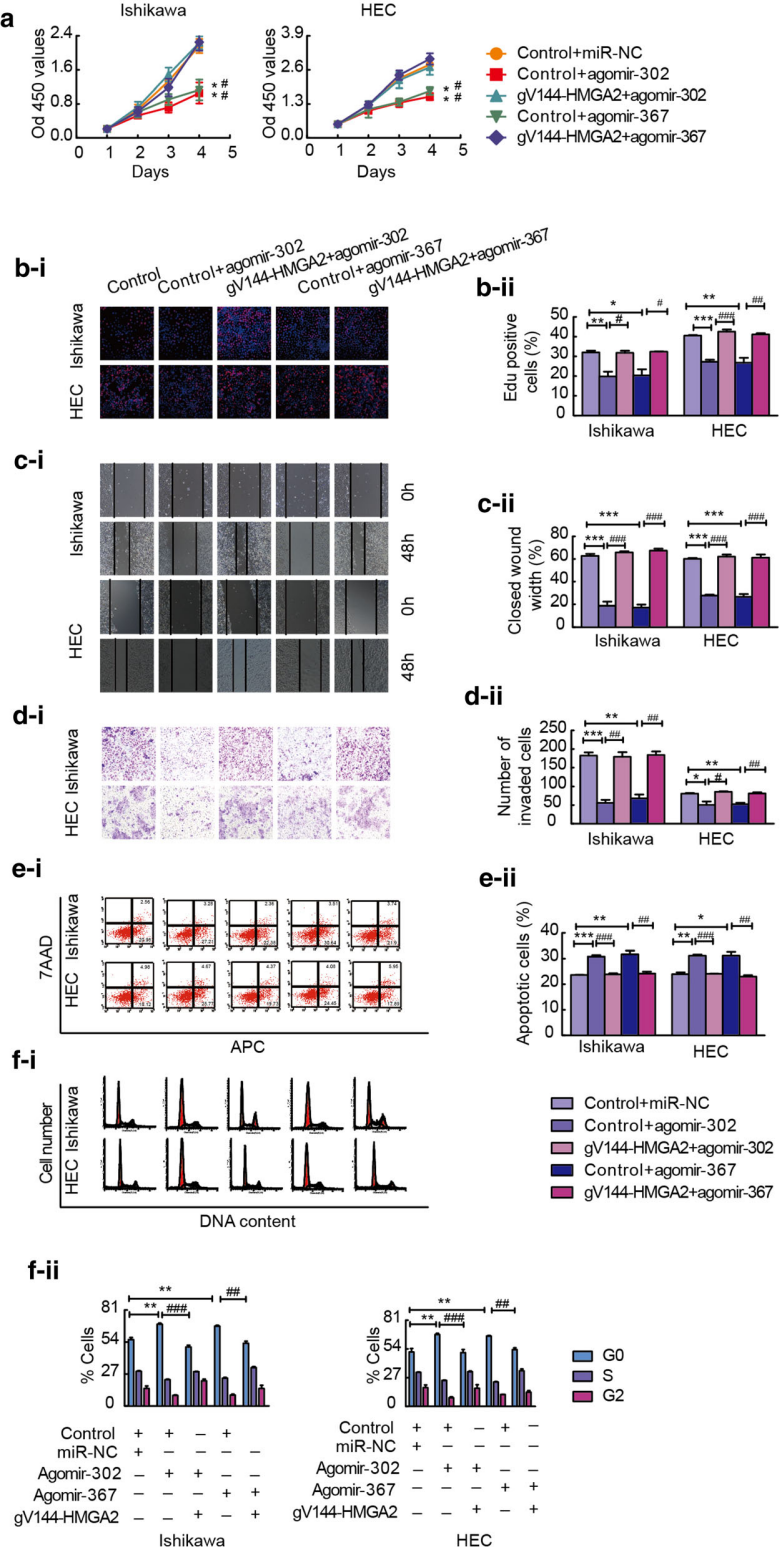
miR-302a-5p/367-3p suppresses the malignant behaviour of endometrial carcinoma cells by targeting HMGA2. **a** and **b** CCK-8 and EdU assays were used to detect proliferation in Ishikawa and HEC-1A cell lines after co-transfection with miR-302a-5p/367-3p and HMGA2-expression constructs. **c** A wound healing assay was performed to investigate the migratory ability of the cells. **d** Quantification of cell invasion in the groups with different expression levels of miR-302a-5p/367-3p and HMGA2. Representative images and statistical plots are presented. **e** Flow cytometric analysis of apoptosis in Ishikawa and HEC-1A cells expressing various levels of miR-302a-5p/367-3p and HMGA2. **f** Representative flow diagrams and graphs showing cell cycle distribution in the groups with different levels of miR-302a-5p/367-3p and HMGA2. Data are presented as the mean ± SEM, (*n* = 3). *P < 0.05, ** *P* < 0.01 vs. control vector + miR-NC, #*P* < 0.05, ## *P* < 0.01, ### *P* < 0.0001 vs. control vector + agomir-302a-5p/367-3p

To verify whether miR-302a-5p/367-3p controls the HMGA2-mediated regulation of the malignant behaviour of endometrial carcinoma cells, we investigated the exact contribution of the miR-302a-5p/367-3p-HMGA2 axis on Ishikawa and HEC-1A cells. Ishikawa and HEC-1A cells were co-transfected with a miR-302a-5p/367-3p overexpressing plasmid and gv144-HMGA2. The cells were divided into the following groups: control vector + miR-NC, control vector + agomir-302a-5p/367-3p and gV144-HMGA2 + agomir-302a-5p/367-3p. The efficiency of HMGA2 re-expression was determined by western blot (Additional file 14: Figure S5). When cells were co-transfected with gV144-HMGA2 and agomir-302a-5p/367-3p, HMGA2 attenuated the suppressive effect of miR-302a-5p/367-3p on cell proliferation, invasion, and migration and prevented apoptosis and cell cycle arrest in the G0/G1 phase. Therefore, the rescue experiments implied that HMGA2 is a functional target of miR-302a-5p/367-3p and ectopic expression of HMGA2 can reverse the anti-tumour effect of miR-302a-5p/367-3p (Fig. 6a-f).

### Overexpression of miR-302a-5p/367-3p combined with knockdown of HMGA2 significantly inhibits tumour growth in vivo

**Fig. 7:**
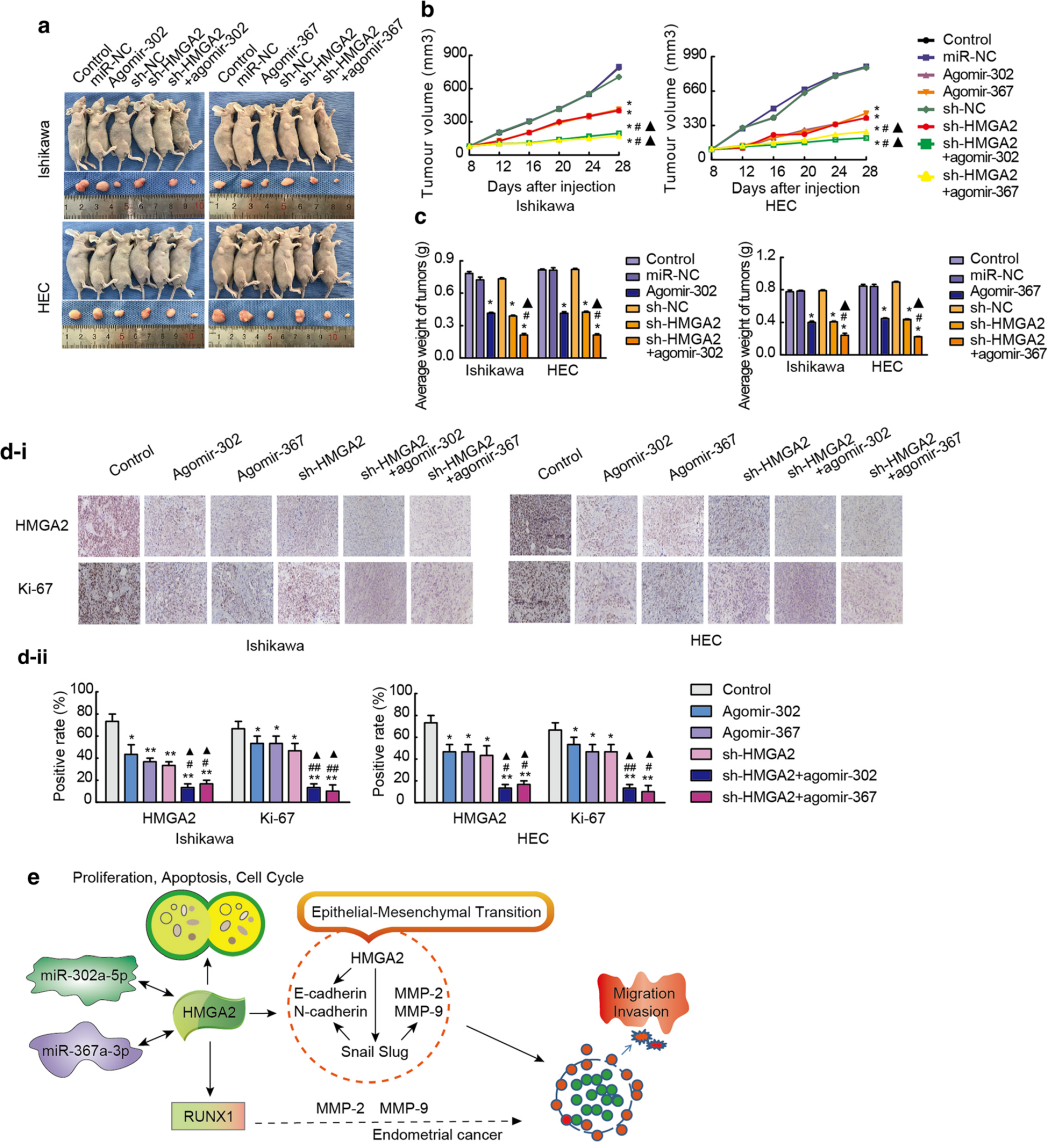
In vivo tumour xenografts. **a** Nude mice bearing tumours composed of cells from the respective groups; sample tumours from each group are shown. **b** and **c** Tumour volume was measured every 4 days after injection. Twenty-eight days later, the mice were sacrificed, and the tumours were excised and weighed. *P < 0.001 vs. the control group, #*P* < 0.001 vs. the agomir group, ▲*P* < 0.001 vs. the sh-HMGA2 group. **d** Expression levels HMGA2, Ki-67. Positive cells are stained brown. Quantified protein levels (area %) in representative xenograft tumours from each treatment group are shown. Data are presented as the mean ± SEM. (n = 3 per group). *P < 0.05, ** *P* < 0.01, ***P < 0.0001 vs. the control group, #*P* < 0.05, ## *P* < 0.01, ### *P* < 0.0001 vs. the agomir group, ▲*P* < 0.05, ▲▲ *P* < 0.01, ▲▲▲*P* < 0.0001 vs. the sh-HMGA2 group. **e** A working model showing the interaction between miRNAs and HMGA2 in Ishikawa and HEC-1A cells

To determine the function of HMGA2 and miR-302a-5p/367-3p in vivo, we analysed the effects of miR-302a-5p/367-3p overexpression or HMGA2 knockdown and their combinatorial effect on endometrial tumour xenografts in nude mice. Ishikawa and HEC-1A cells were co-transfected with a miR-302a-5p/367-3p-overexpressing plasmid and sh-HMGA2. The cells were divided into the following groups: control, miR-NC, agomir-302a-5p/367-3p, sh-NC, sh-HMGA2, and sh-HMGA2 + agomir-302a-5p/367-3p. The expression of miR-302a-5p/367-3p was determined by qRT-PCR, and the expression of HMGA2 was determined by qRT-PCR and western blotting. The results showed that the expression of HMGA2 was the lowest in the sh-HMGA2 + agomir-302a-5p/367-3p group and the expression of miR-302a-5p/367-3p was the highest in the Ishikawa and HEC-1A cells (Additional file 15: Figure S6). In vivo, the volume and weight of tumours were lower in the sh-HMGA2 group than in the sh-NC group. Additionally, the tumours in the agomir-302a-5p/367-3p group were smaller than those in the miR-NC group. In particular, tumours were the smallest in the group co-transfected with sh-HMGA2 with agomir-302a-5p/367-3p (Fig. 7a-c). According to the immunohistochemical assessment, relative to the agomir-302a-5p/367-3p and sh-HMGA2 groups, the expression of HMGA2 and Ki-67 was the lowest in the co-transfected group. In addition, the expression of EMT-related proteins including MMP-2, MMP-9, Snail, Slug, N-cadherin, and Ki-67 was lowest in the co-transfected group, whereas the expression of E-cadherin was the highest (Additional file 15: Figure S6).

## Discussion

Our study demonstrated that high expression of HMGA2 correlates with poor clinical outcomes in endometrial cancer and that its expression is significantly correlated with clinical stage, tumour grade, and metastasis. Overexpression of HMGA2 promoted proliferation, migration, and invasion and inhibited apoptosis and cell cycle arrest in endometrial cancer cells, indicating that HMGA2 plays an oncogenic role in endometrial carcinoma. The results of this study provide compelling evidence on the function and role of HMGA2 in endometrial cancer.

miRNAs are involved in physiological processes such as cell differentiation, apoptosis, proliferation, embryonic development, and stem cell renewal. miRNAs are used as potential biomarkers in the detection and prognosis of cancer, with broad clinical applications [20]. Most miRNAs hybridize to complementary bases on the 3′-untranslated region (3’-UTR) of target mRNAs, leading to the negative regulation of target gene expression via translational inhibition or mRNA degradation [21]. Accumulating evidence has confirmed the abnormal expression of miRNAs in endometrial cancer [22]. miR-302a-5p/367-3p, predicted by bioinformatics software (TargetScan and miRanda), can bind to the 3’-UTR of HMGA2 as demonstrated by the dual-luciferase reporter assay. We demonstrated that the expression level of HMGA2 was decreased after overexpression of miR-302a-5p/367-3p in endometrial carcinoma cell lines. Additionally, the expression of miR-302a-5p/367-3p was detected after artificially altering the levels of HMGA2. On the one hand, miR-302a-5p/367-3p was found to regulate HMGA2 in a unidirectional manner; on the other hand, HMGA2 was found to regulate miR-302a-5p/367-3p expression. Therefore, we concluded that there is a regulatory feedback loop between miR-302a-5p/367-3p and HMGA2. The concept of feedback regulation of miRNAs by their target genes is widely accepted [23, 24]. In previous studies, the miR-302 family was shown to exert anti-tumour effects in several cancers, including liver, cervical, ovarian and gastric cancers [25–28]. However, the function of miR-302-5p/367-3p in endometrial carcinoma cell lines has rarely been studied. In this study, we demonstrated that the expression of miR-302-5p/367-3p is low in endometrial carcinoma tissues and that miR-302a-5p/367-3p expression is associated with muscular invasion and metastasis. We also demonstrated that low expression of miR-302a-5p/367-3p is correlated with poor outcomes in endometrial cancer. In endometrial cancer cell lines, high levels of miR-302-5p/367-3p can inhibit malignant behaviours of cells.

Metastasis is one of the important reasons behind the high mortality rate among cancer patients. Therefore, understanding the mechanism of invasion and metastasis in endometrial carcinoma may provide the basis for molecular therapy to inhibit progression of endometrial cancer and enable the discovery of novel ways to treat and prevent cancer metastasis. EMT is characterized by a decrease in the expression of cellular adhesion molecules such as E-cadherin and an increase in the expression of intercellular proteins such as N-cadherin, resulting in a mesenchymal cell morphology. Alterations in the expression of these proteins reduce cell-cell adhesion, leading to the proliferation of cancer cells and EMT, which is a key process in cancer metastasis [29, 30]. Transcriptional regulators of the SNAIL family, including Snail and Slug, act as key transcription factors to regulate EMT by directly binding to a specific E-box on the E-cadherin promoter. The expression of Snail and Slug is an early event and plays a central role in EMT [31]. The induction of EMT by MMPs has been well characterized in a variety of cancer cells [32], and it involves transmembrane protein regulation, including the release of the extracellular domain of E-cadherin and a reduction in adhesion. MMP-2 and MMP-9 have been found to be associated with invasive endometrial cancers [33]. HMGA2 is widely considered a driving factor of EMT in tumours; additionally, it is known that miRNA-mediated regulation of target genes can lead to reversal of EMT [34, 35]. In this study, the overexpression of miR-302a-5p/367-3p or knockdown of HMGA2 in endometrial cancer cells decreased the expression of EMT-related proteins, such as MMP-2, MMP-9, Snail, Slug and N-cadherin, while increasing the expression of E-cadherin. Thus, we confirmed that in endometrial cancer cells, HMGA2 is a functional target of miR-302a-5p/367-3p in the regulation of EMT-associated proteins. HMGA2, as an important regulatory factor for cancer cell metastasis, can directly affect the expression of EMT-related transcription factors, including Snail and Slug during the EMT process [36]. In addition, HMGA2 can regulate other genes and influence the processes of metastasis. Researchers have analysed the differences in gene expression profiles of tumour and non-tumour endometrial tissues and demonstrated the overexpression of RUNX1 in invasive endometrioid carcinoma tissues [37, 38]. A study by Planagumà et al. demonstrated the association of MMP-2 and MMP-9 with RUNX1 [39]. An orthotopic mouse model of endometrial cancer demonstrated that RUNX1 promotes distant metastasis [40]. Our study demonstrated that overexpressed of RUNX1 increased the expression of MMP-2 and MMP-9 in Ishikawa and HEC-1A cells. The results indicated that RUNX1 promotes distant metastasis in endometrial carcinoma at least partially dependent on the regulation of MMP-2 and MMP-9 expression. HMGA2 can regulate the transcription of several genes by enhancing or inhibiting transcription factor recruitment and affecting the expression of genes via protein-DNA or protein-protein interactions [41, 42]. We retrieved and analysed the data from the TCGA dataset to determine the relationship between the protein levels of HMGA2 and RUNX1 in endometrial carcinoma, and the results indicated that the expression of RUNX1 was positively correlated with the expression of HMGA2. Furthermore, a co-immunoprecipitation assay demonstrated that HMGA2 was bound to RUNX1 protein. In addition, overexpression of HMGA2 increased the expression of RUNX1 at the mRNA and protein levels, whereas knockdown of HMGA2 had the opposite effect. Since HMGA2 is an important gene expression modulator in cancer, we proposed that, through regulation of HMGA2, miR-302a-5p/367-3p also indirectly influences RUNX1 protein expression. Then, we confirmed that HMGA2 functions as a bridge to link miR-302a-5p/367-3p and RUNX1. Therefore, miR-302a-5p/367-3p likely regulates extracellular matrix remodelling during invasion and distant metastasis in endometrial cancer by regulating the miR-302a-5p/367-3p-HMGA2 axis.

We further investigated the effect of the binding of miR-302a-5p/367-3p to HMGA2 on the malignant behaviour of endometrial carcinoma in vitro and found that the overexpression of HMGA2 attenuated the suppressive effect of miR-302a-5p/367-3p on the malignant behaviour of endometrial cancer cells. This result indicated that miR-302a-5p/367-3p regulates the malignant behaviour of endometrial carcinoma cells and is at least partially dependent on the regulation of HMGA2. From the assay used to determine the function of HMGA2 and miR-302a-5p/367-3p in vivo, we found that the overexpression of miR-302a-5p/367-3p combined with the knockdown of HMGA2 effectively reduced the volume and weight of transplanted tumours in nude mice. We further observed that the expression of HMGA2 was lowest in cell lines and nude mouse tissues, while the expression of miR-302a-5p/367-3p was highest in the sh-HMGA2 + agomir-302a-5p/367-3p group in cell lines. These results suggest that sh-HMGA2 combined with agomir-302a-5p/367-3p can interfere with HMGA2 expression and the expression of EMT-related proteins including MMP-2, MMP-9, Snail, Slug, N-cadherin to a maximum degree. In addition, knockdown of HMGA2 expression, increased the expression of miR-302a-5p/367-3p. Besides HMGA2, miR-302a or miR-367-3p may target multiple genes and pathways to inhibit tumour growth and metastasis [43, 44]. This indicates that the combination of agomirs and target genes may be more effective in the inhibition of tumour growth in vitro. It has been reported that in glioblastoma, agomir-101 combined with sh-KLF6 can reduce the size of tumours in nude mice more effectively than transfection of either agomir-101 or sh-KLF6 alone and that the survival rate of nude mice is highest in the co-transfection group [45].

## Conclusions

In summary, this study demonstrated that miR-302a-5p/367-3p mediated the ability of HMGA2 to regulate the malignant behaviour of endometrial carcinoma cells. A working model is shown in Fig. 7e. In the future, potential molecular therapies targeting the miRNA/HMGA2 axis should be explored as novel strategies to treat endometrial cancer.

## Additional files

Additional file 1: Table S1. The shRNA clone and the agomir and antagomir sequences. (DOCX 17 kb)

Additional file 2: Table S2. Primer sequences for qRT-PCR. (DOCX 14 kb)

Additional file 3: Table S3. Primary antibodies used for the detection of protein expression. (DOCX 14 kb)

Additional file 4: Table S4. S4-i: The expression of HMGA2 protein in normal endometrial tissue (*n* = 19) and in endometrial carcinoma tissue (*n* = 80); S4-ii: Association between HMGA2 protein expression and the clinicopathologic characteristics of endometrial cancer patients. (DOCX 16 kb)

Additional file 5: Table S5. Association between HMGA2 mRNA expression and the clinicopathologic characteristics of endometrial cancer patients (*n* = 40). (DOCX 16 kb)

Additional file 6: Figure S1. **a** The baseline expression of HMGA2 in four endometrial cancer cell lines by qRT-PCR. **b** and **c** qRT-PCR and western blot analyses were used to determine the transfection efficiency of HMGA2. Data are presented as the mean ± SEM (*n* = 3 per group). *P < 0.05, ** *P* < 0.01, *** *P* < 0.0001. (PDF 507 kb)

Additional file 7: Figure S2. We employed TargetScan and miRanda bioinformatics databases to predict miRNAs that can bind to HMGA2. In total, 25 miRNAs were screened via co-transfection with wild-type dual-luciferase vector-mediated HMGA2 constructs to identify the most suitable miRNAs. Data are presented as the mean ± SEM (*n* = 3 per group). *P < 0.05, ** *P* < 0.01, *** *P* < 0.0001. (PDF 800 kb)

Additional file 8: Figure S3. qRT-PCR was used to determine the transfection efficiency of the miRNAs. Data are presented as the mean ± SEM (*n* = 3 per group). *P < 0.05, ** *P* < 0.01, *** *P* < 0.0001. (PDF 426 kb)

Additional file 9: Table S6. Association between miR-302a-5p expression and the clinicopathologic characteristics of endometrial cancer patients (*n* = 40). (DOCX 15 kb)

Additional file 10: Table S7. Association between miR-367-3p expression and the clinicopathologic characteristics of endometrial cancer patients (*n* = 40). (DOCX 15 kb)

Additional file 11: Table S8. S8-i: The expression of miR-302a-5p in normal endometrial tissue (*n* = 19) and in endometrial carcinoma tissue (*n* = 80); S8-ii: Spearman’s rank correlation analysis of the correlations between expression of miR-302a-5p and HMGA2 protein in endometrial cancer patients (*n* = 80). S8-iii: Association between miR-302a-5p expression and the clinicopathologic characteristics of endometrial cancer patients (*n* = 80). (DOCX 17 kb)

Additional file 12: Table S9. S9-i: The expression of miR-367-3p in normal endometrial tissue (*n* = 19) and in endometrial carcinoma tissue (*n* = 80); S9-ii: Spearman’s rank correlation analysis of the correlations between the expression of miR-367-3p and HMGA2 protein in endometrial cancer patients (*n* = 80). S9-iii: Association between miR-367-3p expression and the clinicopathologic characteristics of endometrial cancer patients (*n* = 80). (DOCX 18 kb)

Additional file 13: Figure S4. **a** and **b** The expression level of RUNX1 in endometrial carcinoma tissues. **c** and **d** The expression of RUNX1 was positively correlated with the expression of HMGA2 in endometrial carcinoma tissue. **e** to **g** Overexpressed of RUNX1 increased the expression of MMP-2 and MMP-9 in Ishikawa and HEC-1A cells. **h** A co-immunoprecipitation assay was used to validate HMGA2 was bound to RUNX1 in Ishikawa and HEC-1A cell lines. Data are presented as the mean ± SEM (*n* = 3 per group). *P < 0.05, ** *P* < 0.01, *** *P* < 0.0001. (PDF 1149 kb)

Additional file 14: Figure S5. The expression of HMGA2 was determined by western blot. Data are presented as the mean ± SEM (*n* = 3 per group). *P < 0.05, ** *P* < 0.01, *** *P* < 0.0001. (PDF 476 kb)

Additional file 15: Figure S6. **a** The expression of miR-302a-5p/367-3p was determined by qRT-PCR in Ishikawa and HEC-1A cells. **b** HMGA2 expression was determined by qRT-PCR and western blotting in Ishikawa and HEC-1A cells. Data are presented as the mean ± SEM (*n* = 3 per group). **c** Expression of EMT-related proteins including MMP-2, MMP-9, Snail, Slug and N-cadherin were detected via immunohistochemistry. *P < 0.05, ** *P* < 0.01, ***P < 0.0001 vs. the control group, #*P* < 0.05, ## *P* < 0.01, ### *P* < 0.0001 vs. the agomir group, ▲*P* < 0.05, ▲▲ *P* < 0.01, ▲▲▲*P* < 0.0001 vs. the sh-HMGA2 group. (PDF 6632 kb)

## Abbreviations

3’-UTR: 3′-untranslated region
AUC: Area under the curve
ELISA: Enzyme-linked immunosorbent assay
EMT: Epithelial-mesenchymal transition
FBS: Fetal bovine serum
HMGA2: High mobility group AT-hook 2
ISH: In situ hybridization
miRNAs: MicroRNAs
MMP-2: Matrix metalloproteinase-2
MMP-9: Matrix metalloproteinase-9
NC: Negative control
NS: Non-significance
PVDF: Polyvinylidene difluoride
qRT-PCR: Quantitative real-time PCR
ROC: Receiver operating characteristic
RUNX1: Runt related transcription factor 1
SDS-PAGE: Sodium dodecyl sulfate-polyacrylamide gel electrophoresis
TCGA: The cancer genome atlas
