# Supplementary material for: miR-302a-5p/367-3p-HMGA2 axis regulates malignant processes during endometrial cancer development
Source: J Exp Clin Cancer Res. 2018 Feb 1;37:19. doi: 10.1186/s13046-018-0686-6 (PMC5796297; doi:10.1186/s13046-018-0686-6)
Supplement: Supplementary file 1 — The shRNA clone and the agomir and antagomir sequences. (DOCX 17 kb) [file 13046_2018_686_MOESM1_ESM.docx]

Additional file 1

Table S1: The shRNA cloned and agomir and antagomir sequences.

| Name | Sequence |
| --- | --- |
| HMGA2-RNAi (4487-1)-a | 5’-  GATCCCCCAAGAGGCAGACCTAGGAAACTCGAG TTTCCTAGGTCTGCCTCTTGGTTTTTGGAT-3’ |
| HMGA2-RNAi (4487-1)-b | 5’-AGCTATCCAAAAACCAAGAGGCAGACCTAGGAAACTCGAGTTTCCTAGGTCTGCCTCTTGGGG-3’ |
| miR-NC | Sense: 5’-UUCUCCGAACGUGUCACGUTT-3’ Antisense: 5’-ACGUGACACGUUCGGAGAATT-3’ |
| Agomir-302a-5p | Sense: 5’-ACUUAAACGUGGAUGUACUUGCU-3’  Antisense: 5’-CAAGUACAUCCACGUUUAAGUUU-3’ |
| Antagomir-302a-5p | Sense: 5’-AGCAAGUACAUCCACGUUUAAGU-3’ |
| Agomir-367-3p | Sense: 5’-AAUUGCACUUUAGCAAUGGUGA-3’  Antisense: 5’-ACCAUUGCUAAAGUGCAAUUUU-3’ |
| Antagomir-367-3p  Agomir-365a-3p  Antagomir-365a-3p  Agomir-1297  Antagomir-1297  Agomir-9-5p  Antagomir-9-5p  miR-15a-5p  miR-16-5p  miR-137  miR-33a-5p  miR-196a-5p  miR-195-5p  miR-376c-3p  miR-370-3p  miR-485-5p  miR-4306  miR-326  miR-302d-3p  miR-190b  miR-548c-3p  miR-488-3p  miR-509-5p  miR-532-3p  miR-450b-5p  miR-708-5p  miR-520e  RUNX1-RNAi –a  RUNX1-RNAi -b | Sense: 5’-UCACCAUUGCUAAAGUGCAAUU-3’  Sense: 5’-UAAUGCCCCUAAAAAUCCUUAU-3’  Antisense: 5’-AAGGAUUUUUAGGGGCAUUAUU-3’  Sense: 5’-AUAAGGAUUUUUAGGGGCAUUA-3’  Sense: 5’-UUCAAGUAAUUCAGGUG-3’  Antisense: 5’-CCUGAAUUACUUGAAUU-3’  Sense: 5’-CACCUGAAUUACUUGAA-3’  Sense: 5’-UCUUUGGUUAUCUAGCUGUAUGA-3’  Antisense: 5’-AUACAGCUAGAUAACCAAAGAUU-3’  Sense: 5’-UCAUACAGCUAGAUAACCAAAGA-3’  Sense: 5’-UAGCAGCACAUAAUGGUUUGUG-3’ Antisense: 5’-AUCGUCGUGUAUUACCAAACAC-3’  Sense: 5’-UAGCAGCACGUAAAUAUUGGCG-3’ Antisense: 5’-AUCGUCGUGCAUUUAUAACCGC-3’  Sense: 5’-UUAUUGCUUAAGAAUACGCGUAG-3’ Antisense: 5’-AAUAACGAAUUCUUAUGCGCAUC-3’  Sense: 5’-GUGCAUUGUAGUUGCAUUGCA-3’ Antisense: 5’-CACGUAACAUCAACGUAACGU-3’  Sense: 5’-UAGGUAGUUUCAUGUUGUUGGG-3’ Antisense: 5’-AUCCAUCAAAGUACAACAACCC-3’  Sense: 5’-UAGCAGCACAGAAAUAUUGGC-3’ Antisense: 5’-AUCGUCGUGUCUUUAUAACCG -3’  Sense: 5’-AACAUAGAGGAAAUUCCACGU-3’ Antisense: 5’-UUGUAUCUCCUUUAAGGUGCA -3’  Sense: 5’-GCCUGCUGGGGUGGAACCUGGU-3’ Antisense: 5’-CGGACGACCCCACCUUGGACCA-3’  Sense: 5’-AGAGGCUGGCCGUGAUGAAUUC-3’ Antisense: 5’- UCUCCGACCGGCACUACUUAAG -3’  Sense: 5’- UGGAGAGAAAGGCAGUA-3’ Antisense: 5’-ACCUCUCUUUCCGUCAU-3’  Sense: 5’-CCUCUGGGCCCUUCCUCCAG-3’ Antisense: 5’- GGAGACCCGGGAAGGAGGUC-3’  Sense: 5’-UAAGUGCUUCCAUGUUUGAGUGU-3’ Antisense: 5’-AUUCACGAAGGUACAAACUCACA -3’  Sense: 5’-UGAUAUGUUUGAUAUUGGGUU-3’ Antisense: 5’-ACUAUACAAACUAUAACCCAA -3’  Sense: 5’-CAAAAAUCUCAAUUACUUUUGC-3’ Antisense: 5’-GUUUUUAGAGUUAAUGAAAACG -3’  Sense: 5’-UUGAAAGGCUAUUUCUUGGUC-3’ Antisense: 5’-AACUUUCCGAUAAAGAACCAG -3’  Sense: 5’-UACUGCAGACAGUGGCAAUCA-3’ Antisense: 5’-AUGACGUCUGUCACCGUUAGU -3’  Sense: 5’-CCUCCCACACCCAAGGCUUGCA-3’ Antisense: 5’- GGAGGGUGUGGGUUCCGAACGU -3’  Sense: 5’-UUUUGCAAUAUGUUCCUGAAUA-3’ Antisense: 5’-AAAACGUUAUACAAGGACUUAU -3’  Sense: 5’-AAGGAGCUUACAAUCUAGCUGGG-3’ Antisense: 5’-UUCCUCGAAUGUUAGAUCGACCC-3’  Sense: 5’-AAAGUGCUUCCUUUUUGAGGG-3’ Antisense: 5’-UUUCACGAAGGAAAAACUCCC-3’  5’-  CACCGACATCGGCAGAAACTAGATGTTCAAGAGACATCTAGTTTCTGCCGATGTCTTTTTTG-3’  5’-  GATCCAAAAAAGACATCGGCAGAAACTAGATGTCTCTTGAACATCTAGTTTCTGCCGATGTC-3’ |
