# Supplementary material for: miR-302a-5p/367-3p-HMGA2 axis regulates malignant processes during endometrial cancer development
Source: J Exp Clin Cancer Res. 2018 Feb 1;37:19. doi: 10.1186/s13046-018-0686-6 (PMC5796297; doi:10.1186/s13046-018-0686-6)
Supplement: Supplementary file 2 — Primer sequences for qRT-PCR. (DOCX 14 kb) [file 13046_2018_686_MOESM2_ESM.docx]

Additional file 2

Table S2: Primer sequences for qRT-PCR.

| Name | Sequence |
| --- | --- |
| HMGA2 | F: CTCAAAAGAAAGCAGAAGCCACTG  R: TGAGCAGGCTTCTTCTGAACAACT |
| has-mir-302a-5p | F: ACTTAAACGTGGATGTACTTGCT |
| has-mir-367-3p  has-mir-365a-3p  has-mir-1297  has-mir-9 | F: GGACTGTTGCTAATATGCAACTC  F: GGTAATGCCCCTAAAAATCCT TAT  F: GCGGTTCAAGTAATTCAG GTG  F: TCTTTGGTTATCTAGCTGTATGA |
| GAPDH | F: GCACCGTCAAGGCTGAGAAC R: TGGTGAAGACGCCAGTGGA |
| U6 | F: CGGGTTTGTTTTGCATTTCT R: AGTCCCAGCATGAACAGCTT |
| RUNX1  MMP-2  MMP-9 | F: TGATGGCTCTGTGGTAGGTG  R: GGCTGGCAATGATGAAAACT  F: ATGACAGCTGCACCACTGAG  R: AGTTCCCACCAACAGTGGAC  F: GTACCACGGCCAACTACGAC  R: GCCTTGGAAGATGAATGGAA |
