# Supplementary material for: miR-302a-5p/367-3p-HMGA2 axis regulates malignant processes during endometrial cancer development
Source: J Exp Clin Cancer Res. 2018 Feb 1;37:19. doi: 10.1186/s13046-018-0686-6 (PMC5796297; doi:10.1186/s13046-018-0686-6)
Supplement: Supplementary file 3 — Primary antibodies used for the detection of protein expression. (DOCX 14 kb) [file 13046_2018_686_MOESM3_ESM.docx]

Additional file 3

Table S3: Primary antibodies used for the detection of protein expression

| Name | Manufacturer | Dilution ratio:  Western blotting, Immunohistochemistry |
| --- | --- | --- |
| HMGA2 | Abcam, Cambridge, UK | 1:1000, 1:400 |
| RUNX1 | Abcam, Cambridge, UK | 1:1000 |
| E-cadherin | Cell Signaling Technology, Inc., Danvers | 1:1000, 1:400 |
| N-cadherin | Cell Signaling Technology, Inc., Danvers | 1:1000, 1:400 |
| MMP-2 | Proteintech, Hangzhou, China | 1:500, 1:200 |
| MMP-9 | Proteintech, Hangzhou, China | 1:500, 1:200 |
| Snail | Proteintech, Hangzhou, China | 1:200, 1:200 |
| Slug | Proteintech, Hangzhou, China | 1:200, 1:200 |
| Ki-67 | Santa Cruz, CA | 1:200 |
| GAPDH | Proteintech, Hangzhou, China | 1:5000 |
