# Supplementary material for: miR-302a-5p/367-3p-HMGA2 axis regulates malignant processes during endometrial cancer development
Source: J Exp Clin Cancer Res. 2018 Feb 1;37:19. doi: 10.1186/s13046-018-0686-6 (PMC5796297; doi:10.1186/s13046-018-0686-6)
Supplement: Supplementary file 4 — S4-i: The expression of HMGA2 protein in normal endometrial tissue (n = 19) and in endometrial carcinoma tissue (n = 80); S4-ii: Association between HMGA2 protein expression and the clinicopathologic characteristics of endometrial cancer patients. (DOCX 16 kb) [file 13046_2018_686_MOESM4_ESM.docx]

Additional file 4

Table S4-i: The expression of HMGA2 protein in normal endometrial tissue and [endometrial carcinoma](javascript:void(0)) tissue

| Group | n | Positive Expression of HMGA2 n(%) | *P* |
| --- | --- | --- | --- |
| Normal endometrial tissue | 2/19 | 10.5% | < 0.0001 |
| [endometrial carcinoma](javascript:void(0)) tissue | 64/80 | 80.0% |  |

Table S4-ii: Association between HMGA2 protein expression and endometrial cancer patients clinicopathologic characteristics.

| Clinical pathological parameters | N | HMGA2 | | *P* |
| --- | --- | --- | --- | --- |
|  |  | (˗) | (+) |  |
| Age |  |  |  | 0.0445* |
| < 60 | 53 | 14 | 39 |  |
| ≥ 60 | 27 | 2 | 25 |  |
| FIGO stage |  |  |  | 0.0209* |
| I | 15 | 5 | 10 |  |
| II | 15 | 5 | 10 |  |
| III | 30 | 3 | 27 |  |
| IV | 20 | 3 | 17 |  |
| Differentiation |  |  |  |  |
| High | 24 | 7 | 17 |  |
| Middle | 32 | 8 | 24 | 0.7275 |
| Low | 24 | 1 | 23 | 0.0201* |
| Muscular invasion |  |  |  | < 0.0001* |
| < 1/2 | 38 | 15 | 23 |  |
| ≥ 1/2 | 42 | 1 | 41 |  |
| Lymphnode metastasis |  |  |  | 0.0159* |
| Negative | 55 | 15 | 40 |  |
| Positive | 25 | 1 | 24 |  |

Note:

FIGO stage: I+II *vs.* III+IV;

Differentiation: *P* = 0.7275, High differentiation *vs.* Middle differentiation; *P* = 0.0201, High differentiation *vs.* Low differentiation
