# Supplementary material for: miR-302a-5p/367-3p-HMGA2 axis regulates malignant processes during endometrial cancer development
Source: J Exp Clin Cancer Res. 2018 Feb 1;37:19. doi: 10.1186/s13046-018-0686-6 (PMC5796297; doi:10.1186/s13046-018-0686-6)
Supplement: Supplementary file 5 — Association between HMGA2 mRNA expression and the clinicopathologic characteristics of endometrial cancer patients (n = 40). (DOCX 16 kb) [file 13046_2018_686_MOESM5_ESM.docx]

Additional file 5

Table S5: Association between HMGA2 mRNA expression and endometrial cancer patients clinicopathologic characteristics

| Clinical pathological parameters |  | N = 40 | HMGA2  Mean ± SD | *P* |
| --- | --- | --- | --- | --- |
| Age | ≥ 60 | 12 | 12.76863 ± 6.217618 | 0.5399 |
|  | < 60 | 28 | 14.20369 ± 6.692881 |  |
| Clinical stage | I + II | 28 | 11.3478 ± 5.296145 | 0.0002* |
|  | III + IV | 12 | 19.43236 ± 5.781095 |  |
| Differentiation | High | 18 | 13.66581 ± 5.89578 |  |
|  | Middle  Low | 12  10 | 13.94481 ±  6.589598  13.76044 ±  4.960453 | 0.9077  0.9723 |
| Infiltration degree | ≥ 1/2 Muscle layer | 6 | 18.92506 ± 6.639181 | 0.0385* |
|  | < 1/2 Muscle layer | 34 | 12.86401 ± 6.144407 |  |
| Lymphnode metastasis | Positive | 7 | 20.59904 ± 4.960453 | 0.0018* |
|  | Negative | 33 | 12.32526 ± 5.948979 |  |
| Vascular invasion | Positive | 5 | 22.99091 ± 2.074509 | 0.0004* |
|  | Negative | 35 | 12.45635 ± 5.924315 |  |
| Distal metastasis | Positive | 2 | 23.37762 ± 0.526545 | 0.0349* |
|  | Negative | 38 | 13.26767 ± 6.367473 |  |

Note:

*P* = 0.9077, High differentiation *vs.* Middle differentiation;

*P* = 0.9723, High differentiation *vs.* Low differentiation.
