# Supplementary figures and images for: miR-302a-5p/367-3p-HMGA2 axis regulates malignant processes during endometrial cancer development
Source: J Exp Clin Cancer Res. 2018 Feb 1;37:19. doi: 10.1186/s13046-018-0686-6 (PMC5796297; doi:10.1186/s13046-018-0686-6)

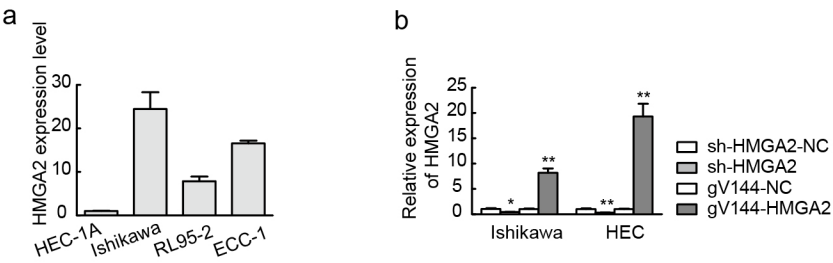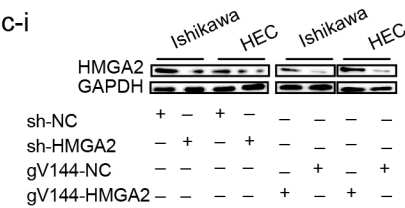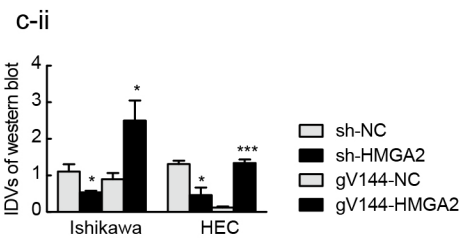

Supplement: Supplementary file 6 — a The baseline expression of HMGA2 in four endometrial cancer cell lines by qRT-PCR. b and c qRT-PCR and western blot analyses were used to determine the transfection efficiency of HMGA2. Data are presented as the mean ± SEM (n = 3 per group). *P < 0.05, ** P < 0.01, *** P < 0.0001. (PDF 507 kb) [file 13046_2018_686_MOESM6_ESM.pdf]

a

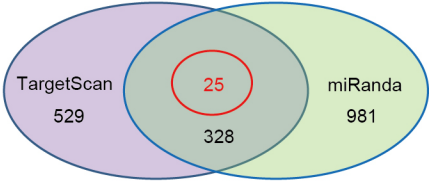

b

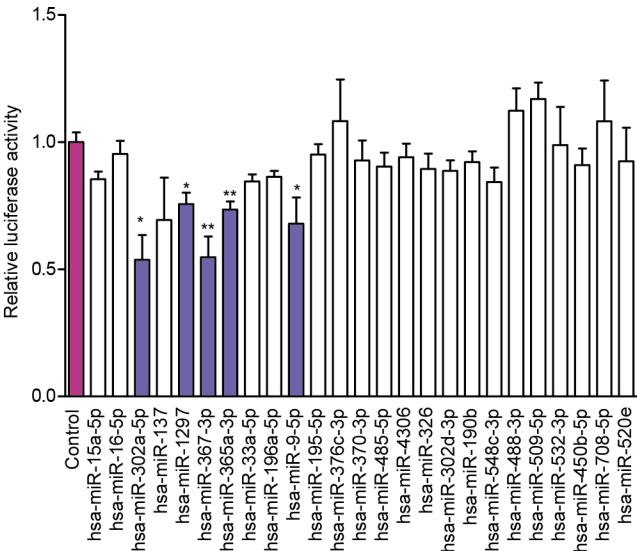

Supplement: Supplementary file 7 — We employed TargetScan and miRanda bioinformatics databases to predict miRNAs that can bind to HMGA2. In total, 25 miRNAs were screened via co-transfection with wild-type dual-luciferase vector-mediated HMGA2 constructs to identify the most suitable miRNAs. Data are presented as the mean ± SEM (n = 3 per group). *P < 0.05, ** P < 0.01, *** P < 0.0001. (PDF 800 kb) [file 13046_2018_686_MOESM7_ESM.pdf]

a-i

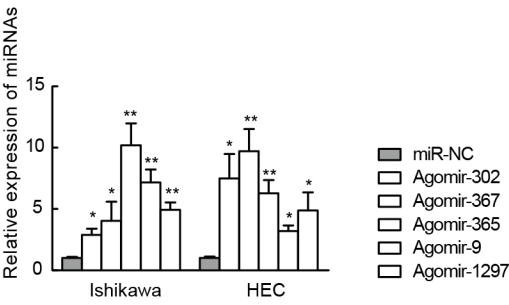

a-ii

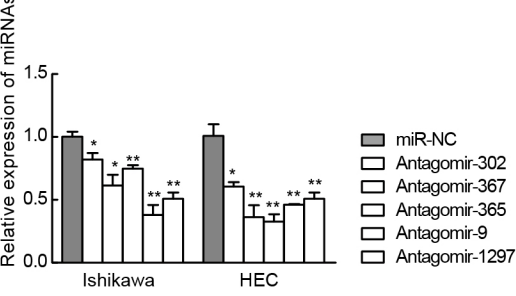

Supplement: Supplementary file 8 — qRT-PCR was used to determine the transfection efficiency of the miRNAs. Data are presented as the mean ± SEM (n = 3 per group). *P < 0.05, ** P < 0.01, *** P < 0.0001. (PDF 426 kb) [file 13046_2018_686_MOESM8_ESM.pdf]

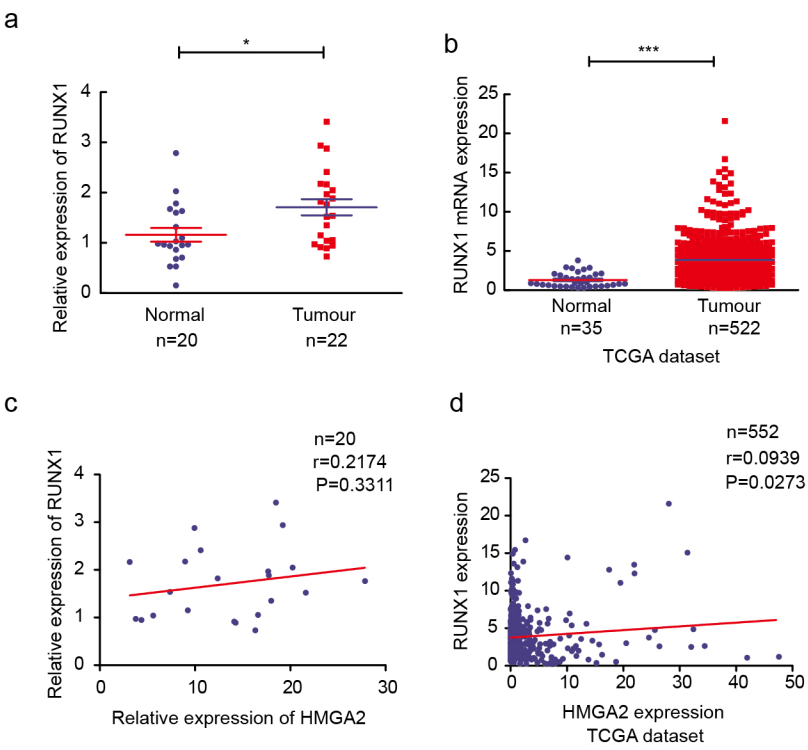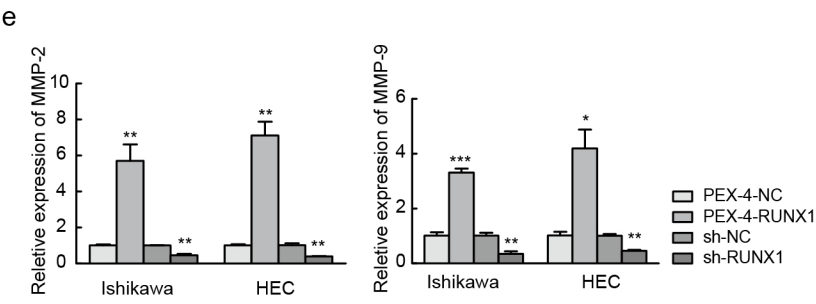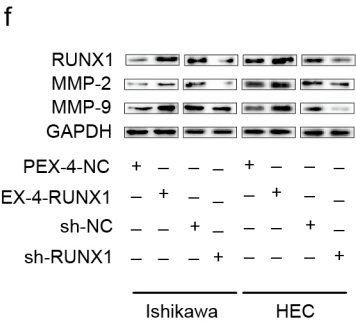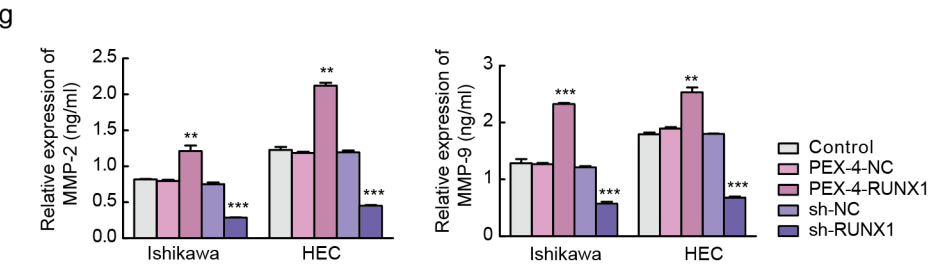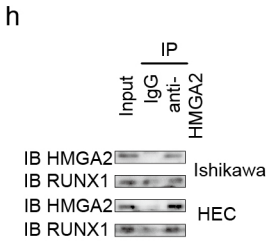

Supplement: Supplementary file 13 — a and b The expression level of RUNX1 in endometrial carcinoma tissues. c and d The expression of RUNX1 was positively correlated with the expression of HMGA2 in endometrial carcinoma tissue. e to g Overexpressed of RUNX1 increased the expression of MMP-2 and MMP-9 in Ishikawa and HEC-1A cells. h A co-immunoprecipitation assay was used to validate HMGA2 was bound to RUNX1 in Ishikawa and HEC-1A cell lines. Data are presented as the mean ± SEM (n = 3 per group). *P < 0.05, ** P < 0.01, *** P < 0.0001. (PDF 1149 kb) [file 13046_2018_686_MOESM13_ESM.pdf]

a-i

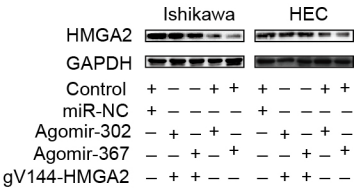

a-ii

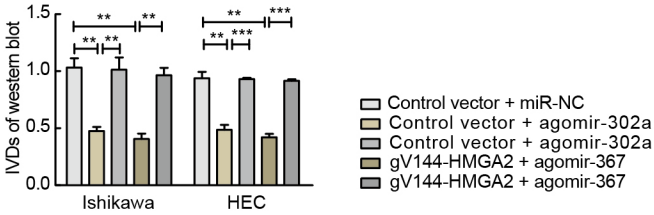

Supplement: Supplementary file 14 — The expression of HMGA2 was determined by western blot. Data are presented as the mean ± SEM (n = 3 per group). *P < 0.05, ** P < 0.01, *** P < 0.0001. (PDF 476 kb) [file 13046_2018_686_MOESM14_ESM.pdf]

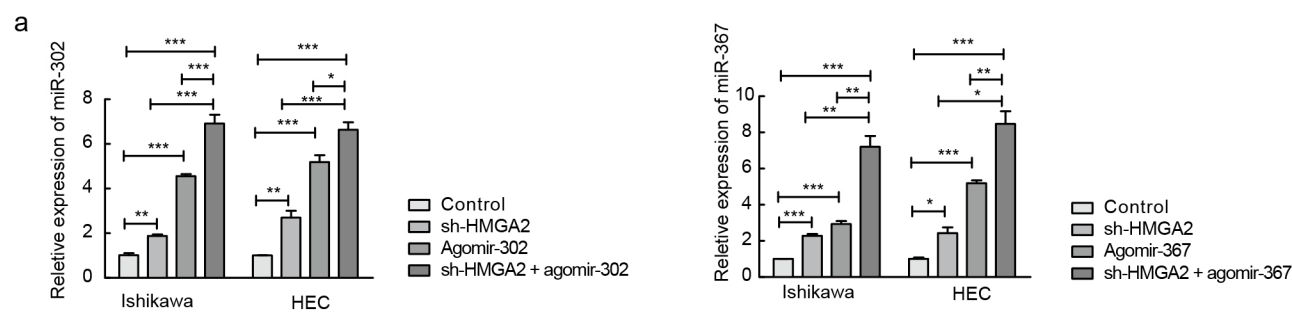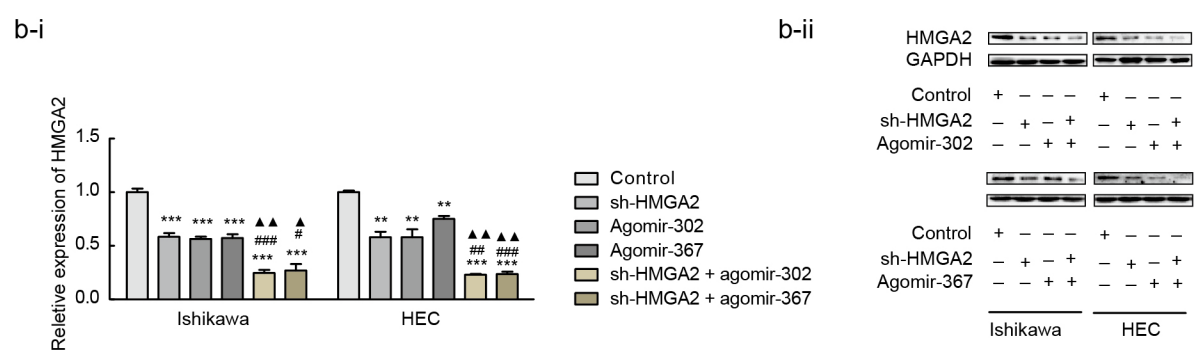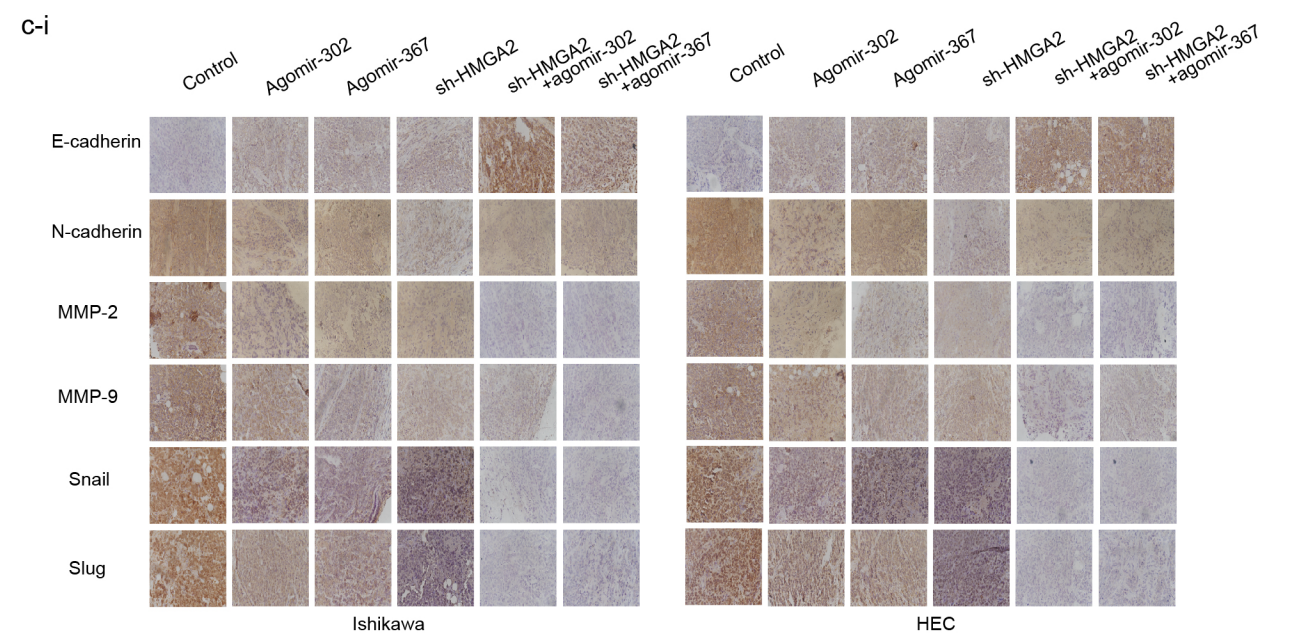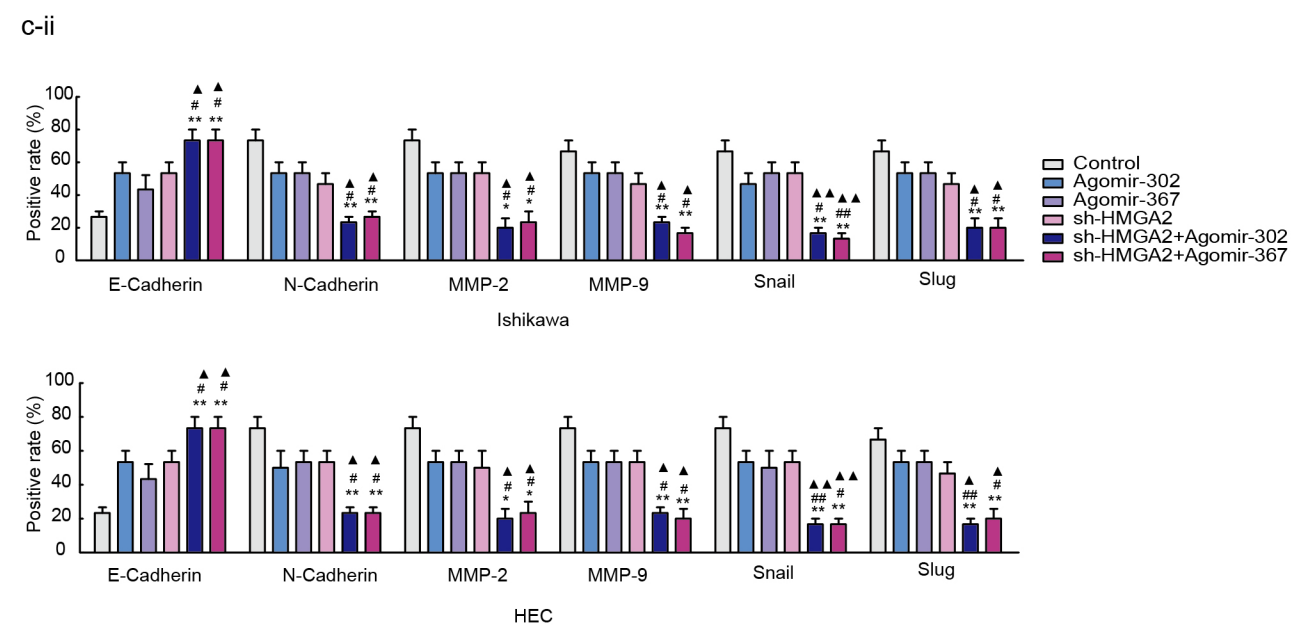

Supplement: Supplementary file 15 — a The expression of miR-302a-5p/367-3p was determined by qRT-PCR in Ishikawa and HEC-1A cells. b HMGA2 expression was determined by qRT-PCR and western blotting in Ishikawa and HEC-1A cells. Data are presented as the mean ± SEM (n = 3 per group). c Expression of EMT-related proteins including MMP-2, MMP-9, Snail, Slug and N-cadherin were detected via immunohistochemistry. *P < 0.05, ** P < 0.01, ***P < 0.0001 vs. the control group, #P < 0.05, ## P < 0.01, ### P < 0.0001 vs. the agomir group, ▲P < 0.05, ▲▲ P < 0.01, ▲▲▲P < 0.0001 vs. the sh-HMGA2 group. (PDF 6632 kb) [file 13046_2018_686_MOESM15_ESM.pdf]
