# Supplementary material for: miR-302a-5p/367-3p-HMGA2 axis regulates malignant processes during endometrial cancer development
Source: J Exp Clin Cancer Res. 2018 Feb 1;37:19. doi: 10.1186/s13046-018-0686-6 (PMC5796297; doi:10.1186/s13046-018-0686-6)
Supplement: Supplementary file 9 — Association between miR-302a-5p expression and the clinicopathologic characteristics of endometrial cancer patients (n = 40). (DOCX 15 kb) [file 13046_2018_686_MOESM9_ESM.docx]

Additional file 9

Table S6: Association between miR-302a-5p expression and endometrial cancer patients clinicopathologic characteristics

| Clinical pathological parameters |  | N = 40 | miR-302a-5p Mean ± SD | *P* |
| --- | --- | --- | --- | --- |
| Age | ≥ 60 | 12 | 0.255059 ± 0.083775 | 0.7532 |
|  | < 60 | 28 | 0.245224 ± 0.08936 |  |
| Clinical stage | I + II | 28 | 0.256454 ± 0.080506 | 0.3754 |
|  | III + IV | 12 | 0.228855 ± 0.100309 |  |
| Differentiation | High | 18 | 0.239044 ± 0.076405 |  |
|  | Middle  Low | 12  10 | 0.25911 ±  0.093532  0.251486±  0.09773 | 0.5392  0.7224 |
| Infiltration degree | ≥ 1/2 Muscle layer | 6 | 0.179346 ± 0.067391 | 0.0381* |
|  | < 1/2 Muscle layer | 34 | 0.26032 ± 0.085393 |  |
| Lymphnode metastasis | Positive | 7 | 0.232454 ± 0.094928 | 0.6131 |
|  | Negative | 33 | 0.251509 ± 0.085889 |  |
| Vascular invasion | Positive | 5 | 0.167591 ± 0.087503 | 0.0284* |
|  | Negative | 35 | 0.259686 ± 0.081631 |  |
| Distal metastasis | Positive | 2 | 0.115948 ± 0.025305 | 0.0291* |
|  | Negative | 38 | 0.255133 ± 0.084375 |  |

Note:

*P* = 0.5392, High differentiation vs. Middle differentiation;

*P* = 0.7224, High differentiation vs. Low differentiation.
