# Supplementary material for: miR-302a-5p/367-3p-HMGA2 axis regulates malignant processes during endometrial cancer development
Source: J Exp Clin Cancer Res. 2018 Feb 1;37:19. doi: 10.1186/s13046-018-0686-6 (PMC5796297; doi:10.1186/s13046-018-0686-6)
Supplement: Supplementary file 10 — Association between miR-367-3p expression and the clinicopathologic characteristics of endometrial cancer patients (n = 40). (DOCX 15 kb) [file 13046_2018_686_MOESM10_ESM.docx]

Additional file 10

Table S7: Association between miR-367-3p expression and endometrial cancer patients clinicopathologic characteristics.

| Clinical pathological parameters |  | N = 40 | MiR-367-3p Mean ± SD | *P* |
| --- | --- | --- | --- | --- |
| Age | ≥ 60 | 12 | 0.183663 ± 0.081816 | 0.0755 |
|  | < 60 | 28 | 0.134169 ± 0.074106 |  |
| Clinical stage | I + II | 28 | 0.161242 ± 0.076477 | 0.1461 |
|  | III + IV | 12 | 0.120491 ± 0.080081 |  |
| Differentiation | High | 18 | 0.150348 ± 0.086113 |  |
|  | Middle  Low | 12  10 | 0.139388 ±  0.079271  0.155045 ±  0.068895 | 0.8796  0.7517 |
| Infiltration degree | ≥ 1/2 Muscle layer | 6 | 0.096608 ± 0.058435 | 0.0848 |
|  | < 1/2 Muscle layer | 34 | 0.158266 ± 0.079483 |  |
| Lymphnode metastasis | Positive | 7 | 0.113486 ± 0.075902 | 0.2043 |
|  | Negative | 33 | 0.156554 ± 0.078554 |  |
| Vascular invasion | Positive | 5 | 0.05745 ± 0.018274 | 0.0052* |
|  | Negative | 35 | 0.162098 ± 0.076549 |  |
| Distal metastasis | Positive | 2 | 0.057148 ± 0.009613 | 0.0996 |
|  | Negative | 38 | 0.153852 ± 0.078927 |  |

Note:

*P* = 0.8796, High differentiation vs. Middle differentiation;

*P* = 0.7517, High differentiation vs. Low differentiation
