# Supplementary material for: miR-302a-5p/367-3p-HMGA2 axis regulates malignant processes during endometrial cancer development
Source: J Exp Clin Cancer Res. 2018 Feb 1;37:19. doi: 10.1186/s13046-018-0686-6 (PMC5796297; doi:10.1186/s13046-018-0686-6)
Supplement: Supplementary file 11 — S8-i: The expression of miR-302a-5p in normal endometrial tissue (n = 19) and in endometrial carcinoma tissue (n = 80); S8-ii: Spearman’s rank correlation analysis of the correlations between expression of miR-302a-5p and HMGA2 protein in endometrial cancer patients (n = 80). S8-iii: Association between miR-302a-5p expression and the clinicopathologic characteristics of endometrial cancer patients (n = 80). (DOCX 17 kb) [file 13046_2018_686_MOESM11_ESM.docx]

Additional file 11

Table S8-i: The expression of miR-302a-5p in normal endometrial tissue and [endometrial carcinoma](javascript:void(0)) tissue.

| Group | n | Positive Expression of miR-302a n(%) | *P* |
| --- | --- | --- | --- |
| Normal endometrial tissue | 16/19 | 84.2% | 0.0015 |
| [endometrial carcinoma](javascript:void(0)) tissue | 35/80 | 43.75% |  |

Table S8-ii: The relationship between the expression of miR-302a-5p and HMGA2 protein.

| miR-302a-5p | HMGA2 | | Total |
| --- | --- | --- | --- |
|  | (˗) | (+) |  |
| (˗) | 3 | 42 | 45 |
| (+) | 13 | 22 | 35 |
| Total | 16 | 64 | 80 |

Table S8-iii: Association between miR-302a-5p expression and endometrial cancer patients clinicopathologic characteristics.

| Clinical pathological parameters | N | miR-302a-5p | | *P* |
| --- | --- | --- | --- | --- |
|  |  | (˗) | (+) |  |
| Age |  |  |  | 0.9288 |
| < 60 | 53 | 30 | 23 |  |
| ≥ 60 | 27 | 15 | 12 |  |
| FIGO stage |  |  |  | 0.0232* |
| I | 15 | 4 | 11 |  |
| II | 15 | 8 | 7 |  |
| III | 30 | 18 | 12 |  |
| IV | 20 | 15 | 5 |  |
| Differentiation |  |  |  |  |
| High | 24 | 10 | 14 |  |
| Middle | 32 | 19 | 13 | 0.1894 |
| Low | 24 | 16 | 8 | 0.0822 |
| Muscular invasion |  |  |  | < 0.0001* |
| < 1/2 | 38 | 12 | 26 |  |
| ≥ 1/2 | 42 | 33 | 9 |  |
| Lymphnode metastasis |  |  |  | < 0.0001* |
| Negative | 55 | 22 | 33 |  |
| Positive | 25 | 23 | 2 |  |

Note:

FIGO stage: I+II *vs.* III+IV;

Differentiation: *P* = 0.1894, High differentiation *vs.* Middle differentiation; *P* = 0.0822, High differentiation *vs.* Low differentiation.
